# BPTF-665aa mediate chromatin remodeling drives chemoresistance in T-LBL/ALL

**DOI:** 10.1186/s13046-025-03556-8

**Published:** 2025-11-07

**Authors:** Rong-Hui Chen, Mei Li, Zhen-Zhong Zhou, Xiao-Jie Fang, Yong Zhu, Yuan Zhang, Xu Liu, Hai-Long Li, Jing Feng, Li-Yan Song, Rong-Min Yu, Tian-Xiao Gao, Xiao-Peng Tian, Wei-Juan Huang

**Affiliations:** 1 Department of Medical Oncology, Sun Yat-sen University Cancer Center, Guangzhou, P.R. China; 2 State Key Laboratory of Oncology in South China, Collaborative Innovation Center of Cancer Medicine, Sun Yat-sen University Cancer Center, Guangzhou, P.R. China; 3 Department of Pharmacology, College of Pharmacy, Jinan University, Guangzhou, P.R. China; 4 Biotechnological Institute of Chinese Materia Medica, Jinan University, Guangzhou, P.R. China; 5 Department of Pathology, Sun Yat-sen University Cancer Center, Guangzhou, P.R. China; 6 Department of General Surgery, The First Affiliated Hospital of Anhui Medical University, Hefei, P.R. China; 7 Department of Radiation Oncology, Sun Yat-sen University Cancer Center, Guangzhou, P.R. China; 8 Department of Nuclear Medicine, Sun Yat-sen University Cancer Center, Guangzhou, P.R. China

**Keywords:** T-LBL/ALL, CircBPTF, BPTF-665aa, Chromatin remodeling, Chemoresistance, Small-molecule inhibitor

## Abstract

Chemoresistance remains a major challenge in addressing T-cell lymphoblastic lymphoma/leukemia (T-LBL/ALL), underscoring the necessity for novel strategies to unravel the molecular factors driving resistance. Through transcriptomic profiling, circBPTF was found to be markedly overexpressed in chemoresistant samples. Further functional experiments demonstrated that BPTF-665aa, the protein product of circBPTF, plays a pivotal role in mediating resistance. Notably, BPTF-665aa prevents the ubiquitination degradation of full-length BPTF, and promotes chromatin accessibility at key promoter sites, such as that of *c-Myc* promter 2 (P2), facilitating transcriptional activation crucial for cellular survival and proliferation under therapeutic stress. Structural studies confirmed the motifs of BPTF-665aa, including the Plant Homeodomain (PHD) finger and Bromodomain, essential for its chromatin remodeling function. HY-B0509 was identified as a small-molecule inhibitor of BPTF-665aa, with molecular docking and dynamics simulations showing stable binding to critical residues within the protein’s active site. Overall, this study introduces a new mechanism where circBPTF affects chromatin accessibility, causing chemoresistance, making BPTF-665aa as a potential therapeutic target for treating T-LBL/ALLs.

**Supplementary Information:**

The online version contains supplementary material available at 10.1186/s13046-025-03556-8.

## Highlights

This study identifies BPTF-665aa, a protein translated from circBPTF, as a key regulator of chromatin accessibility in T-cell lymphoblastic lymphoma/leukemia (T-LBL/ALL). BPTF-665aa promotes chromatin remodeling at oncogenic loci, including the *c-Myc* promoter, facilitating transcriptional activation of resistance-associated genes. Furthermore, we identify a small molecule inhibitor targeting BPTF-665aa, highlighting its potential as a therapeutic strategy. These findings establish a new paradigm linking circRNA-derived proteins and chromatin architecture, positioning BPTF-665aa as a promising therapeutic target.

**Supplementary Information:**

The online version contains supplementary material available at 10.1186/s13046-025-03556-8.

## Introduction

T-cell lymphoblastic lymphoma/leukemia (T-LBL/ALL) is an aggressive malignancy originating from immature T-cell precursors [1, 2]. It accounts for 3%–4% of adult non-Hodgkin lymphomas (NHL) and approximately 40% of pediatric NHL cases, with notably higher rates in Asian populations [3]. Clinically, T-LBL/ALL often manifests as a large anterior mediastinal mass, frequently accompanied by complications such as superior vena cava syndrome (SVCS) [4]. While high-intensity chemotherapy regimens recommended by the National Comprehensive Cancer Network (NCCN) are initially effective [5], therapeutic resistance and relapse affect 40%–60% of patients [6], underscoring the need for further investigation into the molecular mechanisms driving chemoresistance. In addition to recurring genetic mutations in *NOTCH1*, *FBXW7*, and *NRAS/KRAS* [7, 8], recent studies have implicated epigenetic modifications-including DNA methylation, histone alterations, and lncRNAs in tumor progression and resistance [9, 10]. However, the exact roles of these molecules in T-LBL/ALL chemoresistance remain poorly understood.

As the core subunit of the NURF complex, Brodomain PHD finger Transcription Factor (BPTF) sustains non-neoplastic homeostasis by modulating chromatin accessibility (e.g., maintaining mammary epithelial differentiation and c-Myc-driven proliferation) [11–13], while propelling oncogenesis via cancer-specific mechanisms: cooperating with p50 NF-*κ*B to promote COX-2-driven lung tumorigenesis [14]; epigenetically dysregulating the c-Myc/PLCG1/p-ERK axis to confer erlotinib resistance in gastric cancer [15]; and overexpressing to activate Cdc25A-mediated metastasis in colorectal cancer [16]. In T-cell lymphoma, it triggers MAPK signaling to enhance proliferation [17]. Despite BPTF dysregulation being an independent predictor of adverse prognosis, its dynamic interactome in T-LBL/ALL drug resistance remains an underexplored frontier, necessitating integrated multi-omics dissection.

Circular RNAs (circRNAs) have garnered considerable attention as key regulators in cancer, owing to their stable, covalently closed-loop structure that shields them from exonuclease degradation [18]. These molecules perform their functions by sequestering miRNAs, associating with RNA-binding proteins, and serving as blueprints for protein assembly, thereby affecting various oncogenic pathways [19]. For example, circCDYL2 binds directly to the GRB7 protein, preventing its ubiquitination and degradation, thus activating the AKT and ERK pathways and contributing to chemotherapy resistance in HER^2+^ breast cancer cells [20]. Likewise, circLINC-PINT, through the peptide it encodes, inhibits glioblastoma cell proliferation and suppresses oncogene transcriptional elongation by interacting with the PAF1 complex [21]. These instances demonstrate the versatile roles of circRNAs in tumorigenesis and resistance mechanisms. Despite these findings, the exact impact of circRNAs on chemoresistance, particularly in T-LBL/ALL, remains insufficiently understood, emphasizing the necessity for further exploration in this area.

In this study, we investigate circBPTF, a circular RNA derived from the *BPTF* gene, and its role in mediating chemoresistance in T-LBL/ALL. Our results reveal that circBPTF encodes BPTF-665aa, a protein that not only stabilizes the full-length BPTF but also enhances chromatin accessibility at the *c-Myc* promoter 2 (P2) region. This dual role strengthens oncogenic transcriptional programs and contributes to chemotherapy resistance. These findings underscore the distinct ability of circRNA-derived proteins to modulate chromatin dynamics and therapy resistance, positioning both circBPTF and BPTF-665aa as promising therapeutic targets in aggressive hematological cancers. Furthermore, virtual screening led to the identification of HY-B0509, a small molecule that selectively targets BPTF-665aa, presenting a potential strategy to overcome chemoresistance in this aggressive disease.

## Methods

### Patient samples and ethics statement

We studied 166 pediatric T-lymphoblastic leukemia/lymphoma (T-LBL/ALL) cases fulfilling WHO 2022 criteria (> 90% blasts; CD3^+^/CD7^+^/TdT^+^ by Immunohistochemistry) from SYSUCC (*n* = 120) and AHAMU (*n* = 46). Paired samples collected at initial diagnosis and upon relapse from select patients were utilized for subsequent functional experiments. All patients were uniformly treated with the BFM-90 chemotherapy protocol for a minimum of 3 months and all completed the VDLP-CAM induction phase. Chemosensitive patients were defined as those achieving complete remission (CR) lasting > 12 months, while chemoresistance was defined as either failure to achieve CR or relapse within ≤ 3 months. For circRNA discovery, paired chemoresistant (*n* = 4) and chemosensitive (*n* = 4) SYSUCC samples underwent ribosomal RNA-depleted library preparation and sequencing (Illumina NovaSeq, 2 × 150 bp). An independent validation set (*n* = 10 chemoresistant; *n* = 10 chemosensitive SYSUCC samples) was used for immunoblotting of the encoded protein BPTF-665aa using a validated antibody. For the purposes of this study, patient samples were obtained from FFPE blocks, frozen tissues, and flow-sorted cells. Ethics approval (SYSUCC-B2022-521-01) was obtained.

### Cell culture

The SUP-T1, Jurkat and K-562 cell strains were obtained from ATCC and cultured in RPMI-1640 medium containing *L*-glutamine and sodium pyruvate (Gibco, USA), and 10% fetal bovine serum (FBS; Gibco, USA). The cells were incubated in a humidified environment at 37 °C with 5% CO_2_ and passaged every 2–3 days to sustain exponential growth.

### CircRNA expression profiling and validation

RNA was isolated from patient specimens using TRIzol reagent (Thermo Fisher Scientific, USA), following the provided protocol. High-throughput sequencing for circRNA profiling was performed using the CIRI2 pipeline (version 2.0.6) and BWA-MEM for genome alignment to GRCh38 [22]. Divergent primers (forward: 5′-CCAGTGTGCAGAAGTTCTCG-3′; reverse: 5′-TGGACCCACACTTGATGACT-3′) and RNase R digestion (GenStar, China) were applied to confirm circBPTF. Sanger sequencing verified the specific back-splicing junction. CircBPTF levels were quantified by Reverse Transcription Quantitative PCR (RT-qPCR), with GAPDH serving as the internal control. Quantification of expression was carried out with the ΔΔCt approach.

### Insertion of FLAG Tag

The FLAG epitope sequence (5′-GACTACAAAGACGATGACGACAAG-3′) was added upstream of the circBPTF open reading frame (ORF) to enable detection and localization of the BPTF-665aa protein [23].

### Cell survival assay

Cell survival was assessed using a Cell Counting Kit (Novus Biologicals, USA). SUP-T1, Jurkat, and K-562 cells were cultured in 96-well plates at a density of 5 × 10^3^ per well and treated with escalating doses of doxorubicin (Dox; Sigma-Aldrich, USA) for 48 h. The optical density at 450 nm was measured using a plate reader. Cell survival was assessed by comparing to the untreated control group.

### Apoptosis analysis

Apoptotic cell death was evaluated through an Annexin V-FITC/PI apoptosis detection kit (Beyotime, China). SUP-T1, Jurkat, and K-562 cells received Dox for a 24-hour period, then washed twice using ice-cold PBS and labeled by Annexin V-FITC and propidium iodide (PI), in accordance with the provided guidelines. Cellular analysis was conducted using a BD FACSCanto flow cytometer, and the data was analyzed with FlowJo software (version 10.8.1).

### Protein analysis and subcellular localization

Protein lysates were prepared using RIPA buffer containing protease inhibitors (Beyotime, China) and quantified with a BCA Protein Assay Kit (Pierce, USA). Western blotting was performed using an anti-FLAG monoclonal antibody (1:1000 dilution; F1804, Sigma-Aldrich, USA) to detect BPTF-665aa expression. To assess protein stability, cycloheximide (CHX; Sigma-Aldrich, USA) chase assays were conducted. To assess protein degradation, cells were exposed to 100 µg/mL CHX for varying durations (0, 2, 4, and 8 h). Protein band intensities were quantified with the assistance of ImageJ (https://ij.imjoy.io/).

Immunofluorescence staining was used to examine the subcellular localization of BPTF-665aa. To fix the cells, 4% paraformaldehyde (Solarbio, China) was applied, followed by permeabilization with 0.1% Triton X-100 (Amresco, USA). The cells were then exposed to an anti-FLAG antibody, and later treated with Alexa Fluor 594-conjugated secondary antibodies (1:1000 dilution; A-11005, Thermo Fisher Scientific, USA). Nuclei were counterstained with DAPI (Sigma-Aldrich, USA), and images were captured under a fluorescence microscope using appropriate filters. To separate nuclear and cytoplasmic fractions, a Nuclear and Cytoplasmic Extraction Reagent (Thermo Fisher Scientific, USA) was used. Western blot was performed using Lamin B1 and GAPDH as markers for the nucleus and cytoplasm, respectively.

### Lentiviral production and shRNA knockdown

Lentiviral particles were generated by co-transfecting HEK293T cells with an expression plasmid, psPAX2 (packaging vector), and pMD2.G (envelope vector) in a 4:3:1 ratio using polyethylenimine (PEI, Polysciences, USA). The viral supernatants were harvested at 48 and 72 h post-transfection, filtered, and concentrated. For transduction, target cells were treated with lentivirus and 8 µg/mL polybrene (Sigma-Aldrich, USA) and subsequently selected with puromycin (2 µg/mL) for 7 days. Short hairpin RNAs (shRNAs) targeting the back-splicing junction of circBPTF (shCirc-1: 5′-CAGCTAGGCCTTGA-3′, shCirc-2: 5′-TACAGGCCTCAG-3′) were inserted into the pLKO.1 vector.

### Chromatin immunoprecipitation quantitative PCR (ChIP-qPCR)

ChIP-qPCR was used to assess the enrichment of BPTF-665aa, BPTF-665aa, as well as the active histone marks H3K4me3 and H3K27ac, at the *c-Myc* P2 region. SUP-T1 and K-562 cells were crosslinked with 1% formaldehyde for 10 min at room temperature, followed by quenching with 125 mM glycine. Chromatin was fragmented to an average size of 200–500 bp via sonication. Immunoprecipitation was performed using an anti-FLAG antibody (Sigma-Aldrich, USA), with IgG as the negative control. The immunoprecipitated DNA was purified and analyzed by qPCR using primers that specifically amplify the *c-Myc* P2 promoter region (Forward: 5′-AAGGAGGTGGCTGGAAACTT-3′; Reverse: 5′-GCAAATTACTCCTGCCTCCA-3′). Enrichment levels were quantified by normalizing against the input DNA.

### Luciferase reporter assay

Luciferase reporter plasmids carrying the *c-Myc* P2 region were co-transfected with circBPTF constructs (WT, Mut, or ORF) into SUP-T1 and K-562 cells. Luciferase activity was measured using a dual-luciferase reporter assay (Promega, USA), where Renilla luciferase acted as the internal reference.

### Co-Immunoprecipitation (Co-IP)

RIPA buffer containing protease and phosphatase inhibitors was used to extract proteins from the cells. The lysates were incubated overnight with anti-FLAG antibodies bound to protein A/G magnetic beads (Thermo Fisher Scientific, USA). Proteins from immunoprecipitation were resolved by SDS-PAGE and examined through Western blotting. Ubiquitination was assessed with anti-ubiquitin antibodies (Cell Signaling Technology, USA).

### Tumor growth and therapy model

Female BALB/c nude mice, aged 3 to 6 weeks, housed in an environment free of pathogens, with a daily 12-hour light/dark alternation, and had continuous access to both food and water. Subcutaneous injections of 1 × 10^7^ SUP-T1 cells, either carrying circBPTF ORF or control (Ctrl) constructs, were administered. Mice with tumors received intraperitoneal injections of doxorubicin (5 mg/kg; Sigma-Aldrich, USA) every third day for two weeks. Tumor growth was monitored every three days and quantified using the formula: (length × width^2^)/2.

To establish the patient-derived xenograft (PDX) model, a disseminated lymphoma model was generated by inoculating mice via tail vein injection with 1 × 10^7^ primary tumor cells isolated from a T-ALL patient [24–26]. Prior to inoculation, these cells were classified into the high expression and low expression groups based on circBPTF expression levels. Beginning on day 20 post-inoculation, tumor-bearing mice received intraperitoneal injections of doxorubicin (Dox) every third day for a period of two weeks to induce gene knockdown.

All procedures complied with institutional ethical guidelines and were approved by the Ethics Committee of the SYUCC (L102042023050H).

### Virtual screening (VS) and molecular dynamics (MD) simulations

Structural modeling of BPTF-665aa was carried out using SWISS-MODEL, referencing the AlphaFold database template A0A7K7W5R3.1. The homology model generated was subsequently optimized with Schrödinger’s Protein Preparation Wizard. Docking simulations employed Glide software, utilizing small molecules from the MCE 50 K Diversity Library and MCE Bioactive Compound Library (Guangzhou Zuoke Biotechnology Development Co., Ltd). Top-ranking compounds from the docking process were refined further using AutoDockTools (ADT).

MD simulations were conducted in GROMACS (version 2022.5) with the CHARMM36 potential model. Protein-ligand complexes underwent 50 ns of simulation to assess binding affinities, stability, and conformational dynamics.

### Statistical analysis

Data are presented as means ± standard deviations (SD). Statistical significance was assessed using two-tailed Student’s *t*-tests or two-way ANOVA in GraphPad Prism (version 10.3.1). Using the median expression level of circBPTF (5.65) from the training cohort as the cutoff, patients were classified into high and low expression groups. This classification criterion was subsequently validated in an independent cohort. Patient outcomes were evaluated by Kaplan-Meier (KM) survival analysis and Cox proportional hazards models, with a *p* value < 0.05 deemed statistically significant.

### Data availability

ChIP-seq, ATAC-seq, and RNA-seq datasets for the K-562 cell line were retrieved from ChIP-Atla (https://chip-atlas.org), a comprehensive public repository for transcription factor binding profiles. Additionally, high-resolution Hi-C data (GSE237898; resolution) was downloaded from the NCBI GEO database (https://www.ncbi.nlm.nih.gov/geo/), with chromatin interaction maps visualized via the WashU Epigenome Browser (https://epigenomegateway.wustl.edu/browser/; hg38 assembly). The raw sequencing data underlying volcano plots in this study has been deposited in GEO under accession number GSE247590.

## Results

### Characterization of circular structure and functional potential of circBPTF

**Fig. 1:**
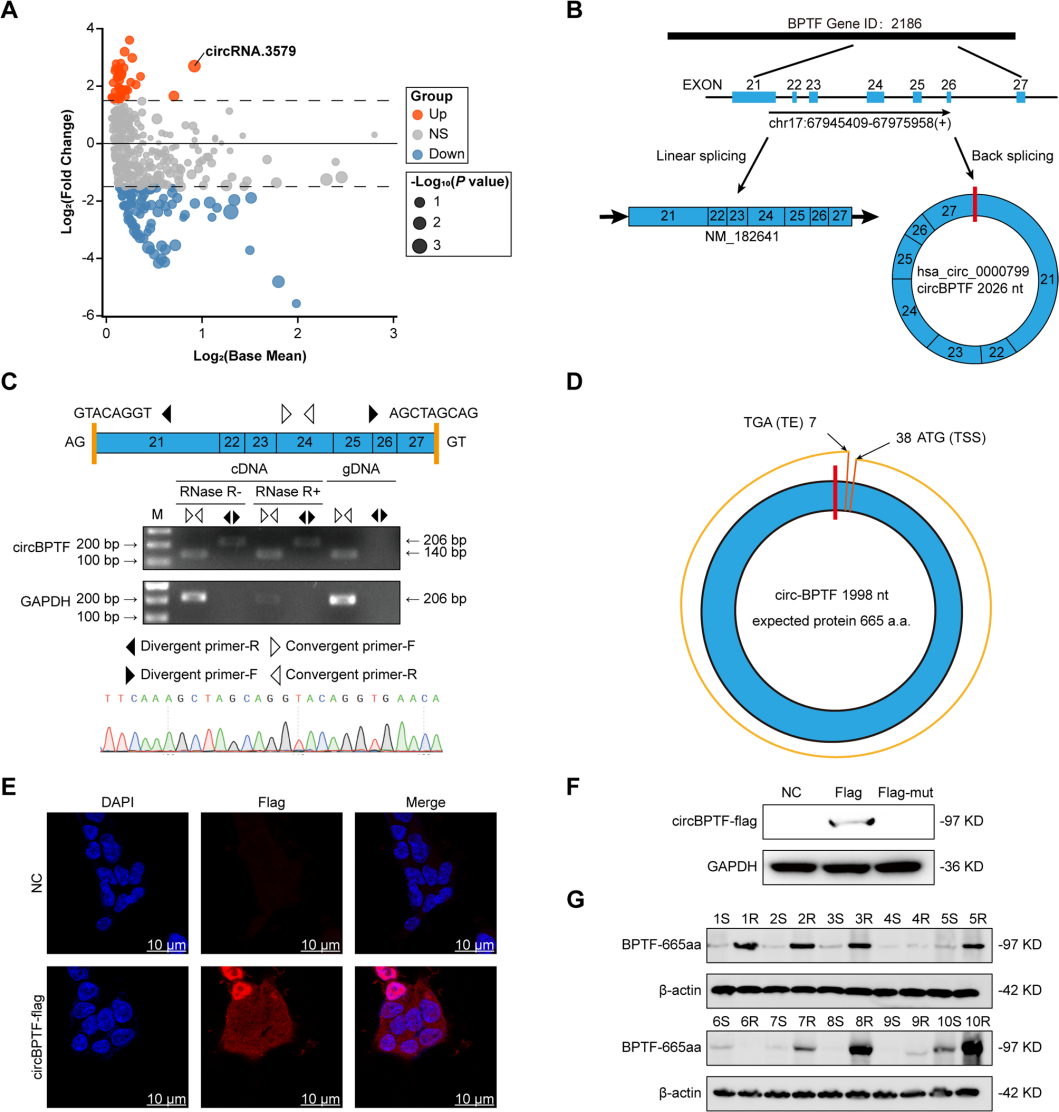
Systematic Characterization and Validation of circBPTF.**(A)** Volcano plot showing differentially expressed circRNAs between drug-resistant and sensitive samples. The x-axis represents log_2_(Base Mean), denoting average expression levels, while the y-axis represents log_2_(Fold Change). Point size correlates with -log_10_(*P*-value). **(B)** Diagram depicting the generation of circBPTF via back-splicing of exons 21–27 from the *BPTF* gene, illustrating the formation of both circular RNA and its linear counterpart (NM_182641). **(C)** Confirmation of the circular nature of circBPTF: Top: Schematic representation of the back-splicing junction connecting exon 27 to exon 21, with the design of divergent and convergent primers highlighted; Middle: Gel electrophoresis results showing successful amplification of circBPTF using divergent primers in RNase R-treated cDNA, while no product was detected in gDNA. GAPDH is used as a loading control; Bottom: Sequencing chromatogram validating the precise exon 27–exon 21 junction characteristic of circBPTF. **(D)** Predicted circular structure of circBPTF, spanning 1998 nucleotides and containing an ORF encoding a 665-amino acid protein. Positions of start (ATG) and stop (TGA) codons are indicated. **(E)** Subcellular distribution of circBPTF in SUP-T1 cells, visualized using FLAG-tagged constructs. Nuclei are counterstained with DAPI. Scale bars represent 10 μm. **(F)** Assessment of the translational potential of circBPTF using FLAG-tagged constructs. Protein expression was analyzed in cells transfected with FLAG-circBPTF and FLAG-mutant constructs. GAPDH serves as the internal control. **(G)** Detection of BPTF-665aa protein expression in drug-resistant (1R–10R) and drug-sensitive (1–10 S) T-LBL/ALL patient samples, with *β*-actin used as the reference protein

To explore the role of circRNAs in drug resistance, eight T-LBL/ALL samples (four resistant and four sensitive) were collected from SYSUCC. Total RNA extraction and high-throughput sequencing were performed. Data processing using the CIRI algorithm identified several differentially expressed circRNAs between resistant and sensitive samples. Notably, circRNA.3579 exhibited notable upregulation in the resistant group (Fig. 1A). CircBase analysis indicated that circRNA.3579 results from back-splicing of the *BPTF* gene, spanning exons 21–27, and generating a 2026-nucleotide circRNA, termed circBPTF (Fig. 1B). Sequencing of the junction between exon 27 and exon 21 confirmed the circular structure, facilitating the primer design for validation.

RNase R digestion and PCR amplification using in vitro-synthesized RNA templates confirmed the circular structure of circBPTF (Fig. 1C). RNase R, an exonuclease that selectively degrades linear RNA while preserving circRNA, efficiently eliminated linear transcripts. PCR using divergent primers detected circBPTF in complementary DNA (cDNA) but not in genomic DNA (gDNA), ruling out genomic contamination. Sanger sequencing verified the back-splicing junction, confirming the circular structure. The Ensembl platform was employed to locate the canonical initiation (ATG) and termination (TGA) codons, while NCBI tools predicted an open reading frame (ORF) of 1998 base pairs that codes for a protein consisting of 665 residues (Fig. 1D).

To assess the translational potential of circBPTF, FLAG-tagged constructs were introduced into cells. Subcellular localization assays revealed that circBPTF primarily localizes to the cytoplasm, supporting its potential for translation (Fig. 1E). Western blotting detected a protein of the expected molecular weight, identified as BPTF-665aa (Fig. 1F). Importantly, BPTF-665aa levels were significantly higher in resistant T-LBL/ALL samples than in sensitive ones (Fig. 1G). Further analysis of patient-derived primary cells revealed significantly elevated expression of both circBPTF and BPTF-665aa in samples after relapse compared to diagnosis, whereas full-length BPTF protein levels showed no significant difference (Supplementary Fig. 1 A, B, Supplementary Fig. 16). Notably, circBPTF expression in T-ALL-derived patient cells was comparable to that observed in T-LBL/ALL-derived cells (Supplementary Fig. 1 C).

### Expression, prognostic value, and role in chemoresistance of circBPTF

**Fig. 2:**
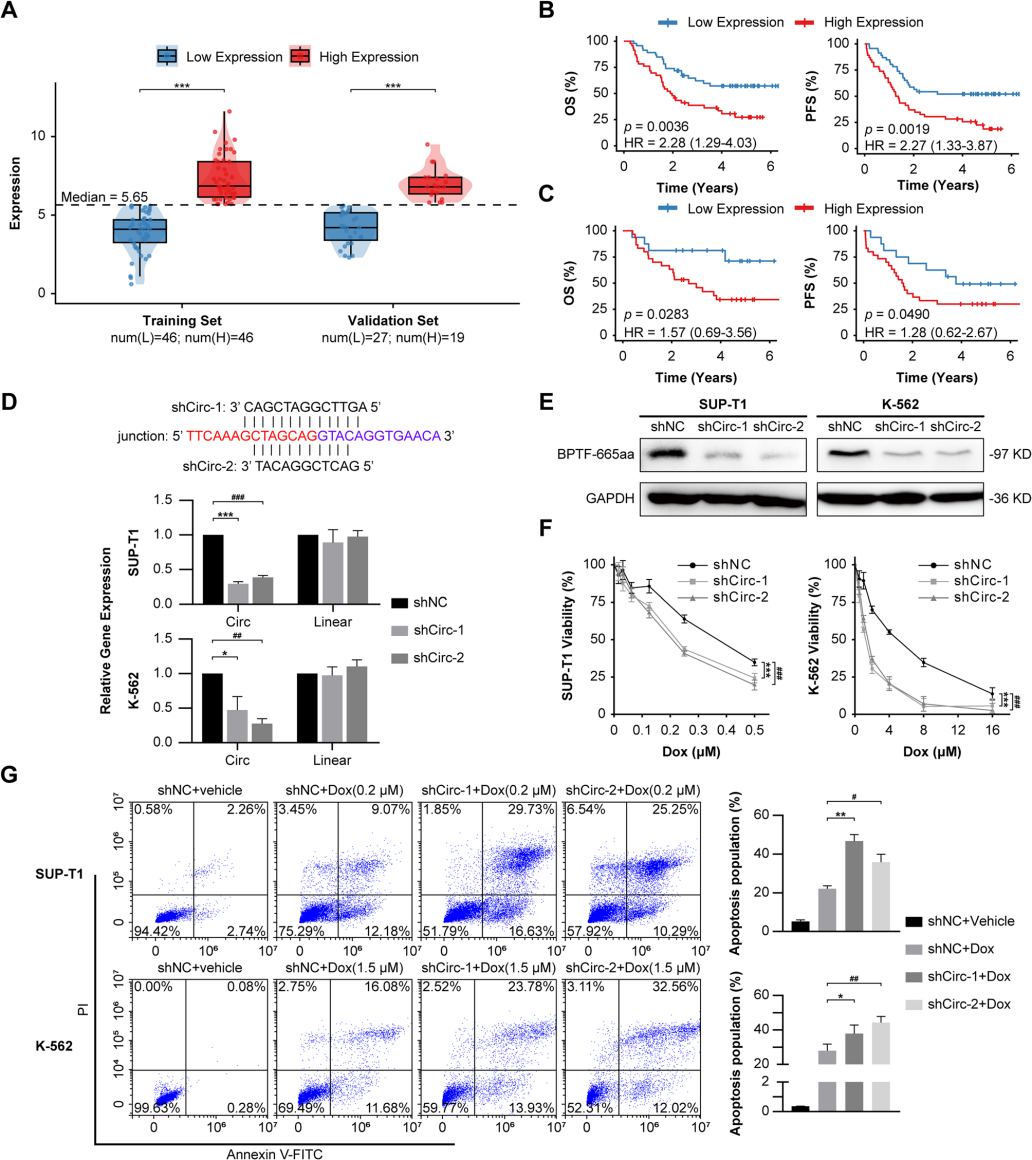
Role of circBPTF in Chemoresistance and Functional Validation.**(A)** Boxplots illustrate the distribution of circBPTF expression in the training and validation cohorts. Patients were stratified into low- and high-expression groups based on the median value (dashed line, Median = 5.65). Group sizes were as follows: training cohort (low: *n* = 46; high: *n* = 46) and validation cohort (low: *n* = 27; high: *n* = 19). Statistical significance was determined by Student’s t-test (***p < 0.001). **(B)**Left: KM survival curves depicting OS in the training set, based on circBPTF expression levels; Right: KM survival curves illustrating PFS in the training set, based on circBPTF expression. Log-rank tests were employed for significance determination. **(C)**Left: KM survival curves depicting OS in the validation set, based on circBPTF expression; Right: KM survival curves illustrating PFS in the validation set, based on circBPTF expression. Log-rank tests were employed for significance determination. **(D)** Verification of circBPTF knockdown efficiency in SUP-T1 and K-562 cells using shRNAs (shCirc-1 and shCirc-2) targeting the back-splicing junction. Relative RNA levels for circBPTF and its linear counterpart were quantified. Data are shown as mean ± SD (*n* = 3). Two-way ANOVA, *p < 0.05, ***p < 0.001 shNC vs. shCirc-1; ^##^*p* < 0.01, ^###^*p* < 0.001 shNC vs. shCirc-2. **(E)** Western blot analysis of BPTF-665aa protein expression in SUP-T1 and K-562 cells following circBPTF silencing. GAPDH was included as the loading control. **(F)** circBPTF knockdown sensitizes SUP-T1 and K-562 cells to Doxorubicin. Data are shown as mean ± SD (*n* = 3). Two-way ANOVA, ***p < 0.001 shNC vs. shCirc-1; ^###^*p* < 0.001 shNC vs. shCirc-2. **(G)** Apoptosis rates in SUP-T1 and K-562 cells subjected to Dox treatment, determined via flow cytometry. Data are shown as mean ± SD (*n* = 3). Student’s *t*-test, *p < 0.05, **p < 0.01 shNC + Dox vs. shCirc-1 + Dox; ^#^*p* < 0.05, ^##^*p* < 0.01 shNC + Dox vs. shCirc-2 + Dox

Immunofluorescence analysis of primary patient samples revealed significantly stronger circBPTF-derived fluorescence signals in therapy-resistant samples compared to therapy-sensitive counterparts (Supplementary Fig. 2). Patients were divided into high and low circBPTF expression groups based on median (5.65), determined from sequencing-derived back-spliced junction read counts (Fig. 2A). The clinicopathological characteristics of these groups are summarized (Supplementary Table S1). KM analysis showed that patients with higher circBPTF expression had significantly shorter overall survival (OS) and progression-free survival (PFS), with hazard ratios (HRs) of 2.28 (*p* = 0.0036) and 2.27 (*p* = 0.0019), respectively (Fig. 2B). These findings were validated in a validation cohort, where elevated circBPTF expression was similarly linked to reduced OS and PFS (HRs: 1.57, *p* = 0.0283 for OS; 1.28, *p* = 0.0490 for PFS; Fig. 2C).

To explore the role of circBPTF in chemoresistance, two shRNA constructs targeting its back-splicing junction (shCirc-1 and shCirc-2) were transfected into K-562, SUP-T1, and Jurkat cells. RT-qPCR analysis showed a marked reduction in circBPTF expression after shRNA treatment, with minimal effect on linear RNA transcripts, thus confirming the specificity of the knockdown strategy (Fig. 2D, Supplementary Fig. 3 A). Additionally, Western blot analysis showed a marked reduction in BPTF-665aa protein levels in cells treated with shCirc-1 and shCirc-2, consistent with the decrease in circBPTF RNA expression (Fig. 2E, Supplementary Fig. 3B).

The effect of circBPTF knockdown on chemoresistance was assessed by determining IC_50_ values for Dox. Silencing circBPTF expression significantly lowered IC_50_ values, indicating increased sensitivity to Dox in both cell lines (Fig. 2F, Supplementary Fig. 3 C). Analysis of apoptosis by flow cytometry showed a marked elevation of apoptotic cells in circBPTF-knockdown samples after Dox treatment, relative to the control group (Fig. 2G, Supplementary Fig. 3D).

### BPTF-665aa exerts a protein-centric role in chemoresistance

**Fig. 3:**
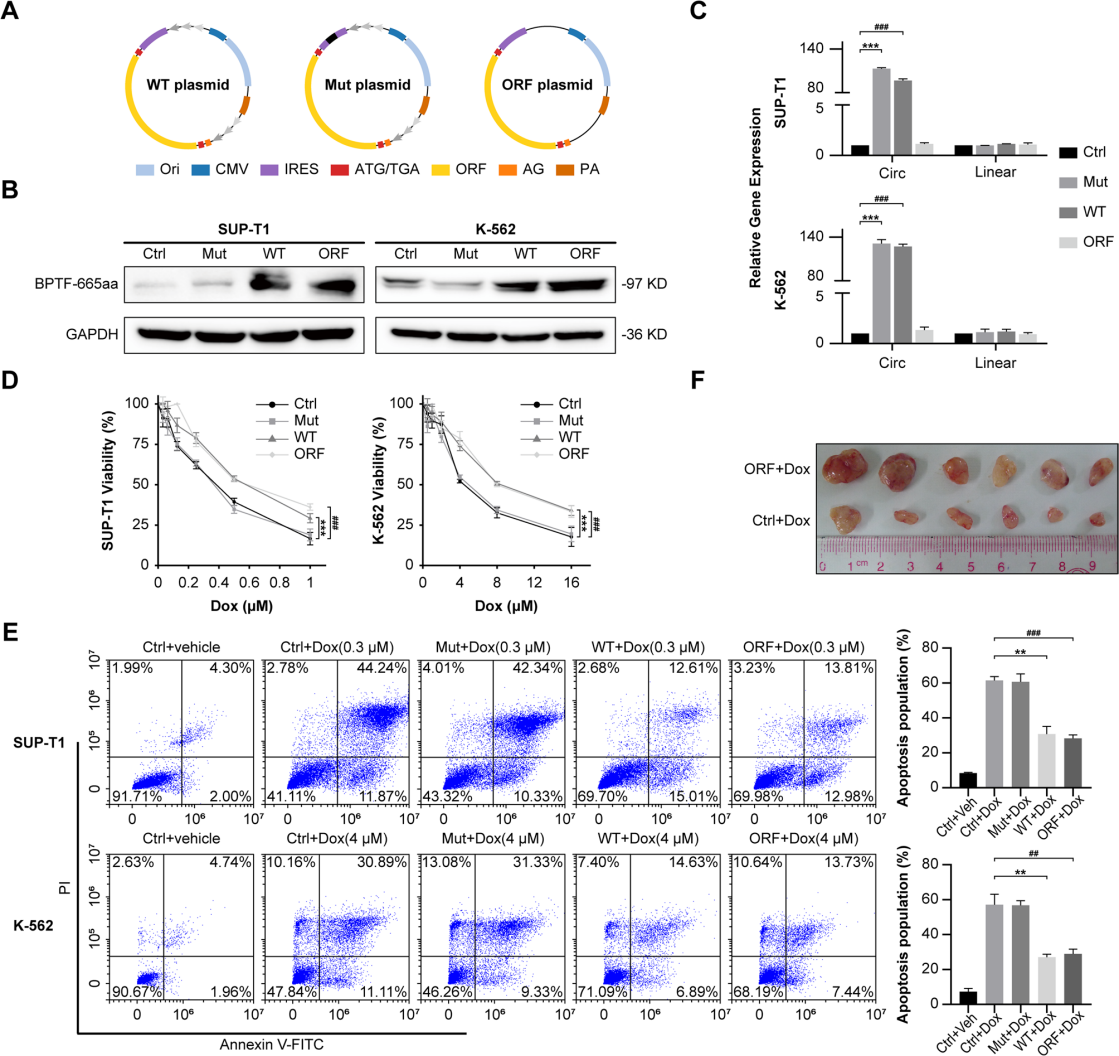
Plasmid Constructs and Functional Validation of circBPTF in Chemoresistance.**(A)** Representation of plasmid designs: WT contains the full-length circBPTF sequence with an intact IRES and ORF; Mut includes a disrupted IRES region that prevents protein translation but permits circular RNA formation; ORF comprises the linear RNA sequence coding for the ORF, without generating circRNA. **(B)** Western blot analysis showing BPTF-665aa protein levels in SUP-T1 and K-562 cells transfected with Ctrl, Mut, WT, or ORF plasmids. GAPDH was used as a reference protein. **(C)** Quantification of circBPTF and linear RNA levels in SUP-T1 and K-562 cells, as measured by RT-qPCR following plasmid transfection. Data are shown as mean ± SD (*n* = 3). Student’s *t*-test, ***p < 0.001 Ctrl vs. Mut; ^###^*p* < 0.001 Ctrl vs. WT. **(D)** Comparison of relative cell viability in SUP-T1 and K-562 cells exposed to Doxorubicin (Dox) after transfection with WT, Mut, ORF, or Ctrl plasmids. Viability is presented relative to untreated controls. Data are shown as mean ± SD (*n* = 3). Two-way ANOVA, ***p < 0.001 Ctrl vs. WT; ^###^*p* < 0.001 Ctrl vs. ORF. **(E)** Apoptosis analysis in SUP-T1 and K-562 cells treated with Dox, assessed by flow cytometry across different plasmid-transfected groups. Data are shown as mean ± SD (*n* = 3). Student’s *t*-test, **p < 0.01 Ctrl + Dox vs. WT + Dox; ^##^*p* < 0.01, ^###^*p* < 0.001 Ctrl + Dox vs. ORF + Dox. **(F)** In vivo xenograft assays measuring tumor growth in mice injected with cells transfected with various plasmid constructs and treated with Dox (5 mg/kg, i.p. every 3 days for 2 weeks). Tumor volumes were monitored and recorded

To investigate the specific roles of circBPTF and its encoded protein BPTF-665aa in chemoresistance, three plasmid constructs were created: WT, Mut, and ORF (Fig. 3A). RT-qPCR showed high circBPTF levels in WT/Mut-transfected cells with equal back-spliced junctions. ORF plasmid (lacking inverted repeats) produced negligible circRNA but still translated BPTF-665aa protein, confirmed by Western blot. We thereby demonstrate the separation of protein translation from circRNA production (Fig. 3B, C, Supplementary Fig. 4 A, B).

To examine the effect of these constructs on drug resistance, *IC*_50_ assays were performed. Cells transfected with the WT plasmid showed significantly higher *IC*_50_ values for Dox compared to those in the Mut or control groups (Fig. 3D, Supplementary Fig. 4 C). Interestingly, Mut-transfected cells expressed circBPTF but lacked BPTF-665aa, resulting in *IC*_50_ values similar to controls. In contrast, ORF-transfected cells, which expressed BPTF-665aa independently of circRNA formation, showed *IC*_50_ values similar to the WT group. Apoptosis assays further supported these findings: WT-transfected cells exhibited significantly lower apoptosis rates under Dox treatment than both Mut and control groups. Similarly, Mut-transfected cells showed apoptosis rates comparable to the controls, whereas ORF-transfected cells exhibited markedly lower apoptosis rates, consistent with BPTF-665aa expression (Fig. 3E, Supplementary Fig. 4D). Additionally, resistance profiling against multiple chemotherapeutics revealed that overexpression of BPTF-665aa significantly increased resistance to Dexamethasone (Dex), L-asparaginase (L-ASP), vincristine (VCR), and daunorubicin (DNR) (Supplementary Fig. 5).

To validate these results in vivo, tumor xenograft experiments were performed using SUP-T1 cells transfected with either ORF or Ctrl plasmids. Following tumor establishment, Dox was administered via intraperitoneal injection. At the endpoint, tumors derived from ORF-transfected cells were significantly larger than those from control cells, confirming the role of BPTF-665aa in chemoresistance (Fig. 3F). Corroboratively, intravenous injection of primary T-ALL cells via tail vein into mice in both high expression and low expression groups revealed that circBPTF upregulation significantly improved Dox efficacy and extended survival (Supplementary Fig. 6, Supplementary Fig. 17).

### Dual roles of BPTF-665aa in protein stability and nuclear regulation

**Fig. 4:**
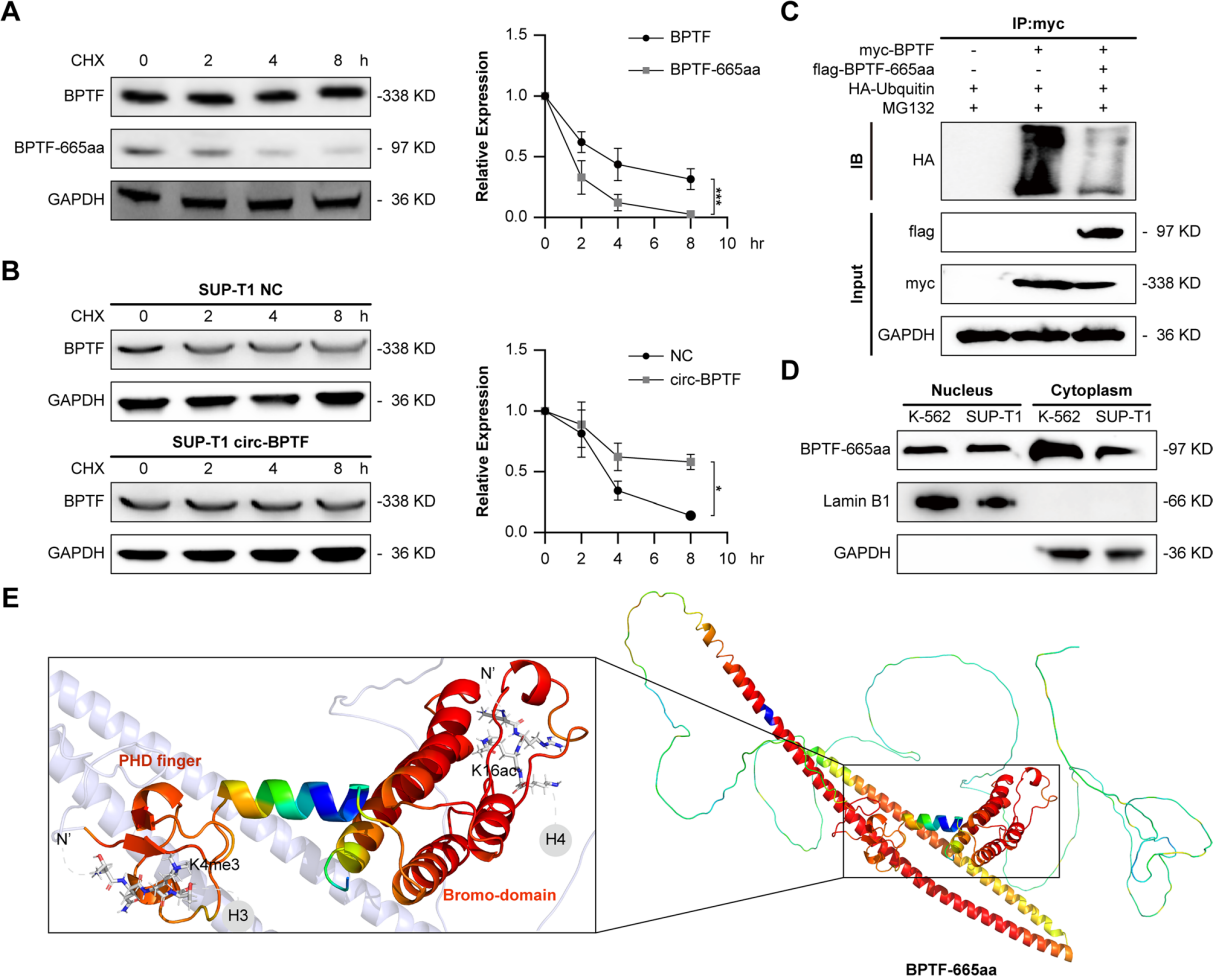
Stability, Localization, and Structural Analysis of BPTF-665aa.**(A)** Cycloheximide (CHX) chase assay evaluating the stability of full-length BPTF and BPTF-665aa in SUP-T1 cells. Protein levels were assessed at 0, 2, 4, and 8 h post-treatment. Data are shown as mean ± SD (*n* = 3). Two-way ANOVA, ***p < 0.001. **(B)** CHX chase assay in circBPTF-overexpressing SUP-T1 cells, measuring the degradation rate of full-length BPTF. Data are shown as mean ± SD (*n* = 3). Two-way ANOVA, *p < 0.05. **(C)** Co-IP assay examining interactions between FLAG-tagged BPTF-665aa and myc-tagged full-length BPTF. MG132 was used to inhibit proteasomal degradation during the assay. **(D)** Subcellular localization of BPTF-665aa in K-562 and SUP-T1 cells, determined by Western blot analysis of nuclear and cytoplasmic fractions. Lamin B1 and GAPDH serve as markers for nuclear and cytoplasmic compartments, respectively. **(E)** Structural modeling of BPTF-665aa was performed using SWISS-MODEL, based on the AlphaFold database template A0A7K7W5R3.1. The predicted structure was analyzed to annotate potential chromatin-associated regions and to model possible interactions with histones

CHX chase assays were employed to compare the stability of BPTF-665aa and full-length BPTF in SUP-T1 cells. Western blot analysis demonstrated that BPTF-665aa had a significantly shorter half-life than the full-length protein, suggesting reduced stability under the experimental conditions (Fig. 4A, Supplementary Fig. 14). When CHX chase assays were performed in SUP-T1 and Jurkat cells overexpressing circBPTF, the degradation of full-length BPTF occurred at a significantly slower rate in these cells compared to controls, indicating that circBPTF stabilizes the full-length protein (Fig. 4B, Supplementary Fig. 7, Supplementary Fig. 15, Supplementary Fig. 18).

To investigate the mechanism behind this stabilization, Co-IP assays were conducted to examine the interactions between BPTF-665aa and full-length BPTF, with a focus on their effects on ubiquitination. Cells were co-transfected with Myc-tagged full-length BPTF and FLAG-tagged BPTF-665aa constructs, followed by immunoprecipitation. The data revealed that BPTF-665aa significantly reduced the ubiquitination of full-length BPTF, suggesting a competitive interaction for the ubiquitination machinery as a possible mechanism (Fig. 4C).

Subcellular fractionation experiments were performed to determine the localization of BPTF-665aa in K-562 and SUP-T1 cells. The protein was found in both the cytoplasm and nucleus. While a reduction in ubiquitination was primarily observed in the cytoplasm, the presence of BPTF-665aa in the nucleus suggests additional roles related to nuclear functions (Fig. 4D).

Structural modeling of BPTF-665aa was carried out using SWISS-MODEL, and the predicted structure was compared with the crystal structure of full-length BPTF (PDB ID: 2F6J). The analysis identified conserved chromatin-binding domains, including the Plant Homeodomain (PHD) finger and Bromo-domain, spanning residues 2867–3014. These domains align with functional elements present in full-length BPTF, further supporting its potential role in chromatin remodeling (Fig. 4E).

### BPTF-665aa-mediated chromatin accessibility at the P2 promoter drives *c-Myc* activation and chemoresistance

**Fig. 5:**
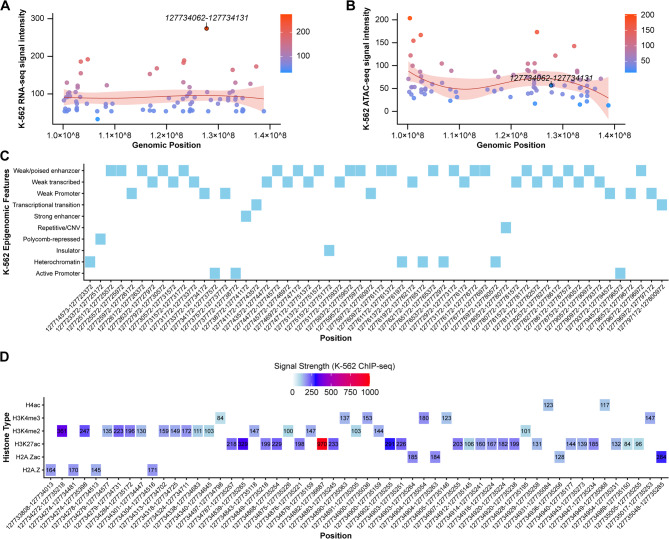
Genomic Landscape and Chromatin Organization of the *c-Myc* Locus. **(A)** RNA-seq signal profile in K-562 cells across the genomic region chr8:100000000–140,000,000, overlaid with BPTF ChIP-seq data to highlight transcriptional activity and BPTF occupancy. **(B)** ATAC-seq signal profile in K-562 cells for the same genomic region, intersected with BPTF ChIP-seq data to assess chromatin accessibility and BPTF binding. (**C**) Chromatin state annotations in K-562 cells across the *c-Myc* region, highlighting active promoters, enhancers, insulators, and heterochromatin. **(D)** Heatmap of histone modification signal strengths in K-562 cells across the *c-Myc* region, including H4ac, H3K4me3, H3K4me2, H3K27ac, H2A.Zac, and H2A.Z marks. Signal intensities are color-coded, with red indicating stronger signals and blue weaker signals

Analysis of publicly available datasets, combined with BPTF ChIP-seq data, revealed a regulatory element within the *c-Myc* locus at chr8:127734062–127,734,131. This region exhibited high transcriptional activity and chromatin accessibility, as confirmed by chromatin state profiling. Enrichment of histone marks like H4ac and H3K4me3, indicative of open chromatin and active transcription, characterized the site as a functional promoter (Fig. 5).

**Fig. 6:**
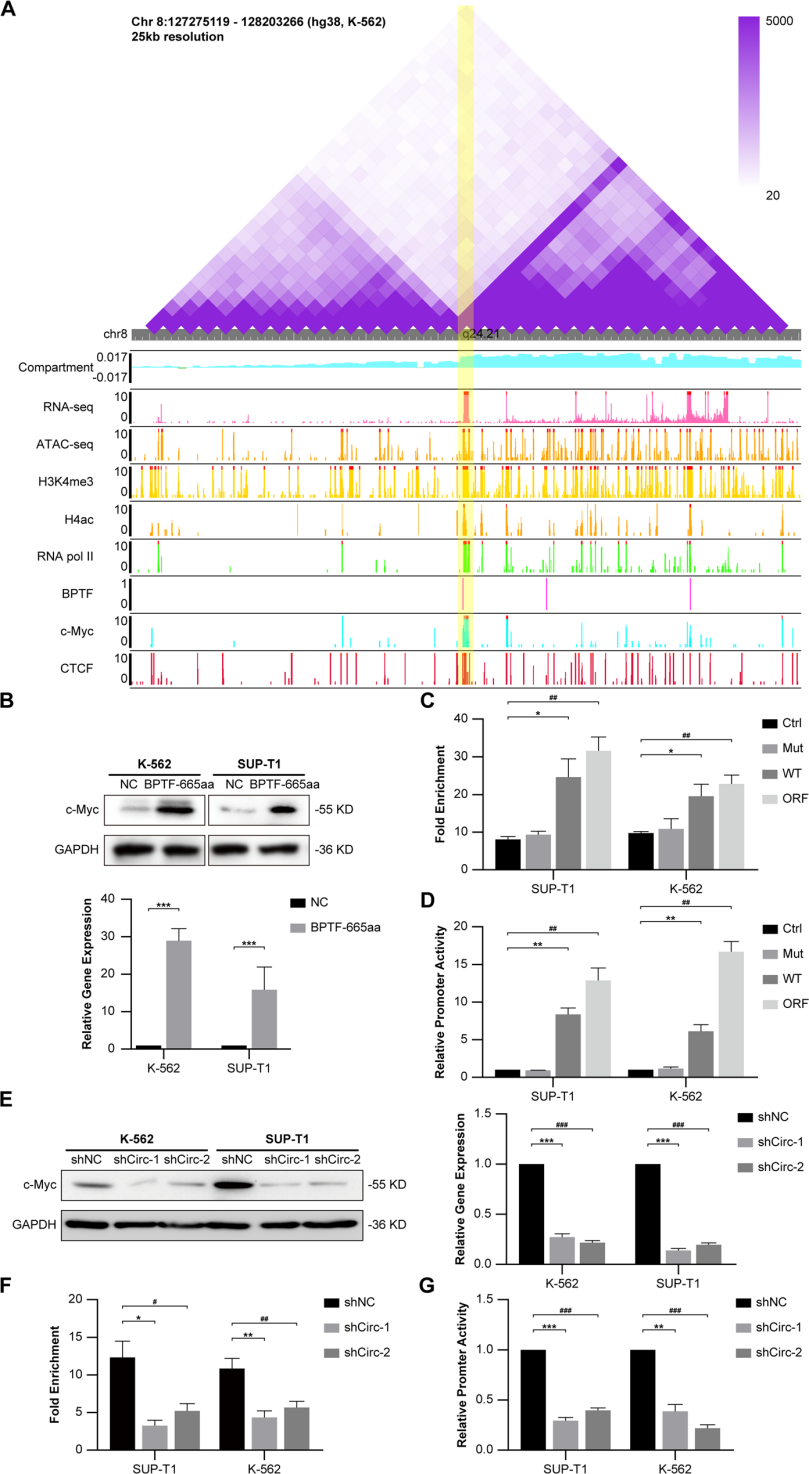
BPTF-665aa and circBPTF Modulate *c-Myc* Expression and Promoter Activity.** (A)** Hi-C contact map of the *c-Myc* locus at 25 kb resolution in K-562 cells, illustrating chromatin interactions and genomic features, including RNA-seq signals, ATAC-seq peaks, histone marks (H3K4me3 and H4ac), RNA polymerase II binding, BPTF occupancy, and CTCF enrichment. **(B) **Top: Western blot analysis of c-Myc protein levels in K-562 and SUP-T1 cells following BPTF-665aa overexpression. GAPDH is used as the loading control; Bottom: RT-qPCR quantification of *c-Myc* mRNA levels in K-562 and SUP-T1 cells overexpressing BPTF-665aa. Data are shown as mean ± SD (*n* = 3). Student’s *t*-test, ***p < 0.001. **(C)** ChIP-qPCR analysis measuring BPTF-665aa binding enrichment at the *c-Myc* P2 promoter. WT contains the full-length circBPTF sequence with an intact IRES and ORF; Mut includes a disrupted IRES region that prevents protein translation but permits circular RNA formation; ORF comprises the linear RNA sequence coding for the ORF, without generating circRNA. Data are shown as mean ± SD (*n* = 3). Student’s *t*-test, *p < 0.05 Ctrl vs. WT; ^##^*p* < 0.01 Ctrl vs. ORF. **(D)** Luciferase reporter assays evaluating *c-Myc* P2 activity in cells transfected with BPTF-665aa constructs. Data are shown as mean ± SD (*n* = 3). Student’s *t*-test, **p < 0.01 Ctrl vs. WT; ^##^*p* < 0.01 Ctrl vs. ORF. **(E) **Left: Western blot analysis of c-Myc protein levels in K-562 and SUP-T1 cells transfected with control (shNC) or circBPTF-targeting shRNAs (shCirc-1 and shCirc-2). GAPDH serves as the loading control; Right: RT-qPCR analysis of *c-Myc* mRNA levels in K-562 and SUP-T1 cells post circBPTF knockdown. Data are shown as mean ± SD (*n* = 3). Student’s *t*-test, ***p < 0.001 shNC vs. shCirc-1; ^###^*p* < 0.001 shNC vs. shCirc-2. **(F)** ChIP-qPCR analysis of BPTF-665aa enrichment at the c-*Myc* P2 promoter post circBPTF knockdown. Data are shown as mean ± SD (*n* = 3). Student’s *t*-test, *p < 0.05, **p < 0.01 shNC vs. shCirc-1; ^#^*p* < 0.05, ^##^*p* < 0.01 shNC vs. shCirc-2. **(G)** Luciferase reporter assay measuring promoter activity of the c-*Myc* P2 promoter post circBPTF knockdown. Data are shown as mean ± SD (*n* = 3). Student’s *t*-test, **p < 0.01, ***p < 0.001 shNC vs. shCirc-1; ^###^*p* < 0.001 shNC vs. shCirc-2

Hi-C analysis showed that the regulatory region at chr8:127734062–127,734,131 lies within a topologically associating domain (TAD) spanning chr8:127,275,119–128,203,266. This TAD is defined by strong chromatin interactions and distinct subregions, including chromatin region B, which plays a crucial role in transcriptional regulation. Chromatin loops within this TAD, anchored by CTCF, position the promoter close to distal enhancers and transcription start sites, facilitating precise control of *c-Myc* transcription. Further analyses confirmed the regulatory role: RNA-seq signals indicated robust transcriptional activity, ATAC-seq data revealed chromatin accessibility, and histone marks like H3K4me3 and H4ac were enriched in the region, consistent with an active promoter state. Additionally, RNA polymerase II occupancy at the promoter indicated active transcriptional engagement. Taken together, these findings emphasize the role of the TAD’s chromatin structure in the regulation of *c-Myc* (Fig. 6A). Concurrently, ATAC-seq showed elevated MYC chromatin accessibility in resistant cells, correlating with enhanced BPTF binding to its promoter (Supplementary Fig. 8).

To validate these findings experimentally, the effect of BPTF-665aa on *c-Myc* transcription was examined. Overexpression of BPTF-665aa in K-562, SUP-T1, and Jurkat cells led to significant elevations in both *c-Myc* RNA and protein levels, as confirmed by RT-qPCR and Western blot analysis (Fig. 6B, Supplementary Fig. 9). These results confirmed that the identified promoter corresponds to the *c-Myc* P2 region [27]. ChIP-qPCR assays showed strong enrichment of BPTF-665aa and the active histone marks H3K4me3 and H3K27ac at the P2 region in cells overexpressing BPTF-665aa, suggesting its direct binding to this promoter (Fig. 6C, Supplementary Fig. 10). Luciferase reporter assays also confirmed these findings by demonstrating increased transcriptional activity of the P2 region in the presence of BPTF-665aa (Fig. 6D). Similar results were obtained through circBPTF overexpression (Fig. 6E, F, G).

### Virtual screening and molecular dynamics simulations identify HY-B0509 as a BPTF-665aa inhibitor

**Fig. 7:**
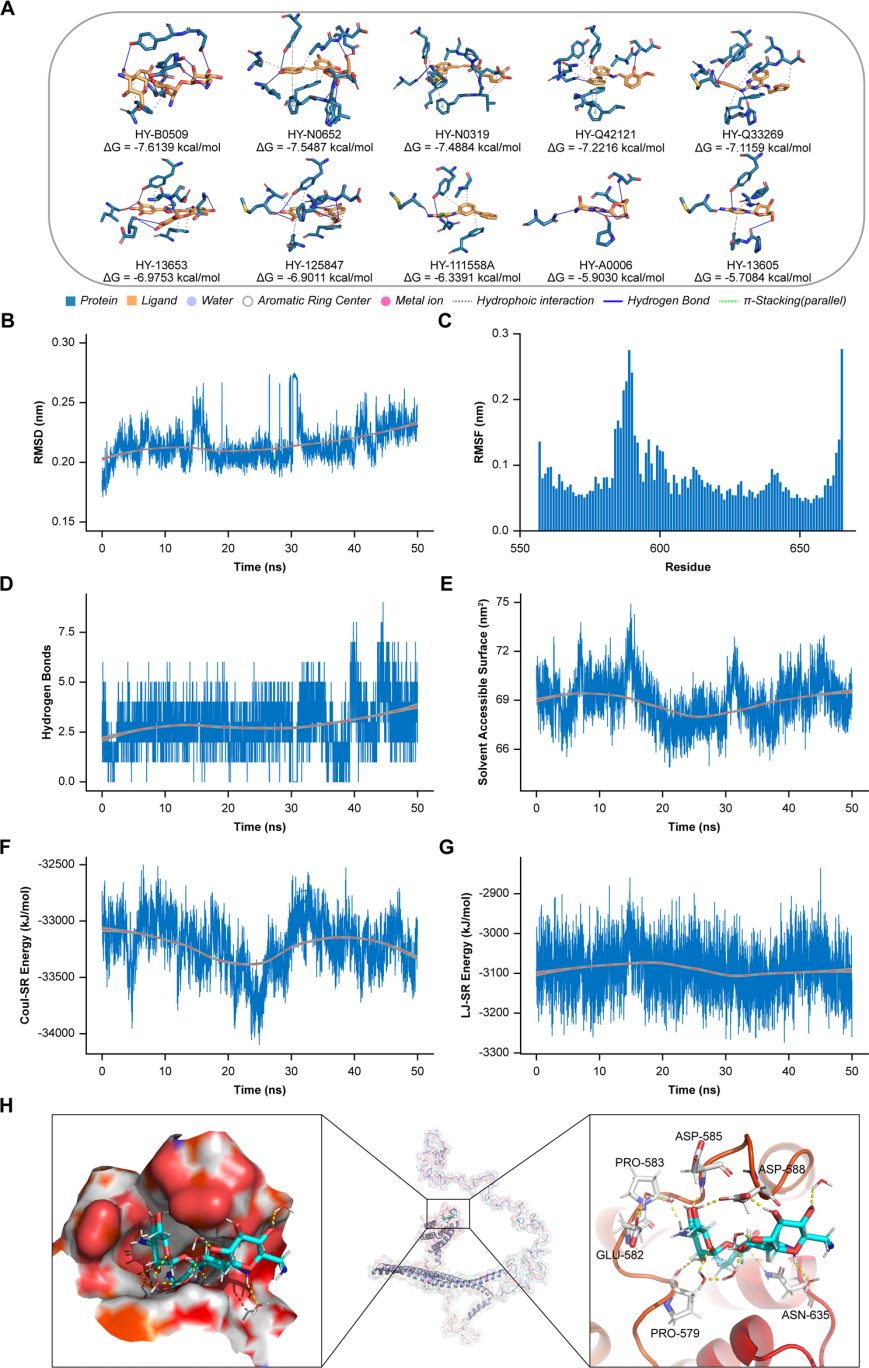
MD Simulation and Interaction Analysis of BPTF-665aa-Ligand Complex.** (A)** Molecular docking analysis of the top 10 compounds targeting BPTF-665aa, ranked by binding free energy (ΔG). **(B)** Protein-ligand RMSD (0–50 ns MD) demonstrating structural equilibration (< 0.3 nm final deviation). **(C)** Residue-specific RMSF peaks (550–600) indicating binding-site flexibility. **(D)** Sustained hydrogen bonding (2.5 ± 0.8 bonds) with transient 4-bond states at 30 ns. **(E)** Solvent-accessible surface area reduction (7.2→6.6 nm²) correlating with binding compaction. **(F)** Coulombic short-range (Coul-SR) energy (−33,500 ± 500 kJ·mol^−1^) showing electrostatic interaction stability. **(G)** Lennard-Jones short-range (LJ-SR) energy (−3,100 ± 100 kJ·mol^−1^) confirming hydrophobic dominance. **(H)** Optimized final binding conformation of the ligand with BPTF-665aa: Left: Surface representation of the protein-ligand interaction. Middle: Positioning of the ligand within the full protein structure. Right: Close-up view highlighting key residues involved in hydrogen bonding and hydrophobic interactions

A VS pipeline was utilized to identify potential inhibitors of BPTF-665aa. The MCE 50 K Diversity Library and the MCE Bioactive Compound Library were used for screening. Key residues in the active binding site, such as ILE596, VAL584, TYR642, and ILE627, were pinpointed through the Sitemap module. Docking simulations, both standard and extra-precision, were performed with Schrödinger Maestro 12.8, narrowing the selection to 50 high-ranking compounds (Supplementary Table S2). To further evaluate the top 10 compounds, 100 docking simulations were carried out with ADT to assess binding conformations and affinities. HY-B0509 emerged as the lead compound, demonstrating the strongest binding affinity with a free energy of ΔG = −7.6139 kcal/mol. Protein-ligand interaction profiling revealed hydrogen bonds with ASP585, ASN635, and GLU582, along with hydrophobic contacts involving PRO583 and PRO579 (Fig. 7A).

To validate the stability of the HY-B0509-BPTF-665aa complex, MD simulations were conducted using GROMACS over a 50 ns trajectory. The stability of the simulated system is supported by comprehensive analyses (Supplementary Fig. 11). Root mean square deviation (RMSD) trajectories remained tightly constrained, with an average fluctuation of ~ 0.2 nm, indicating minimal structural drift throughout the 50 ns simulation (Fig. 7B). Residue-specific root mean square fluctuation (RMSF) profiles highlighted localized flexibility in select regions, though critical binding motifs exhibited restricted mobility (Fig. 7C).

Hydrogen bond interactions between the ligand and protein remained consistently robust, with counts stabilizing within a narrow range (Fig. 7D). Solvent-accessible surface area (SASA) measurements showed no abrupt changes, reflecting sustained structural compactness (Fig. 7E). Nonbonded interaction energies, including Coulombic short-range (Coul-SR) and Lennard-Jones short-range (LJ-SR) terms, displayed moderate fluctuations within thermodynamically stable ranges (Figs. 7F, G). Visualization of the HY-B0509 binding pocket highlighted its spatial positioning within the active site, where hydrogen bonds and hydrophobic interactions aligned closely with key residues (Fig. 7H). Functionally, we experimentally demonstrated that this inhibitor significantly sensitizes resistant cells, effectively mitigating drug resistance (Supplementary Fig. 12).

## Discussion

CircRNAs have been extensively investigated in solid tumors, where they contribute to oncogenesis, immune evasion, and therapy resistance [28, 29]. However, their role in hematological malignancies, particularly in T-LBL/ALL, remains largely unexplored, with few studies addressing their functional significance in this disease. While prior research suggests that circRNAs influence oncogenic pathways in leukemia [30], a systematic characterization of circRNA expression in T-LBL/ALL has not yet been performed. This study bridges this gap by providing the first comprehensive profiling of the circRNA landscape in T-LBL/ALL, identifying distinct expression patterns and unveiling circBPTF as a critical factor in chemoresistance. Moreover, our findings provide strong experimental validation of circBPTF’s circular structure and demonstrate its essential role in chemotherapy resistance, reinforcing the growing recognition of circRNAs as pivotal regulatory molecules in hematological cancers.

Unlike most circRNAs, which exert their functions through miRNA sponging or RNA-binding protein interactions, circBPTF encodes BPTF-665aa, which plays a distinct role in the cytoplasm by stabilizing its full-length counterpart, BPTF. One of its primary functions is preventing BPTF from undergoing ubiquitination and subsequent proteasomal degradation, thereby extending its half-life and maintaining its functional activity. Given BPTF’s well-established role in chromatin remodeling and transcriptional regulation, this stabilization mechanism enhances oncogenic signaling by ensuring the sustained presence of BPTF, reinforcing key transcriptional networks necessary for tumor survival, including those regulated by *c-Myc* [31, 32]. The ability of BPTF-665aa to regulate BPTF stability introduces a previously unrecognized layer of post-translational control exerted by circRNA-derived proteins.

Beyond its cytoplasmic role in protein stabilization, BPTF-665aa exerts a critical nuclear function by directly modulating chromatin accessibility at key oncogenic loci, particularly at the *c-Myc* P2 region, which is essential for the transcriptional activation of chemoresistance genes. Chromatin accessibility plays a fundamental role in regulating gene expression, tumor progression, and therapeutic resistance [12]. However, in contrast to many other circRNAs that primarily exert their effects through cytoplasmic mechanisms, such as miRNA sponging or protein stabilization, circBPTF is the first known circRNA to act by directly modifying chromatin structure. This discovery introduces a novel epigenetic mechanism in which BPTF-665aa enhances chromatin accessibility in the nucleus, providing a mechanism by which circRNA-derived proteins contribute to chemoresistance. In contrast to nuclear circRNAs like EIciRNA, which activate transcription by interacting with RNA polymerase II [33], BPTF-665aa interacts with chromatin remodelers, altering nucleosome positioning to sustain oncogenic transcriptional programs. This unique ability to regulate chromatin and influence transcription in response to chemotherapy stress sets circBPTF apart from other circRNAs. Given the diverse roles circRNAs play in various cancers, acting either as oncogenes or tumor suppressors [34–36], it remains to be explored whether circBPTF follows a similar context-dependent regulatory pattern in other malignancies. To address potential associations with genetic alterations, we specifically analyzed NOTCH1 mutations and FBXW7 mutations. While observable differences emerged, they did not reach statistical significance (Supplementary Fig. 13). Future studies, including functional assays and clinical validation, will be necessary to elucidate its broader role in resistance and therapeutic targeting across different tumor types.

While inhibitors targeting the parental BPTF protein (e.g., BZ1, AU1) have been reported [37, 38], therapeutic strategies against the novel circRNA-encoded BPTF-665aa remain unexplored. To identify potential therapeutic strategies, we leveraged VS followed by MD simulations to discover small-molecule inhibitors targeting BPTF-665aa. This computational approach enabled the efficient screening of an extensive compound library, refining a subset of promising candidates and significantly enhancing the efficiency of the selection process compared to traditional high-throughput screening (HTS) or enzyme-linked immunosorbent assays (ELISA) [39]. Unlike HTS, which primarily evaluates static binding affinity, MD simulations provide dynamic insights into protein-ligand interactions, yielding a more comprehensive assessment of inhibitor stability and specificity [40, 41]. Among the candidates, HY-B0509 exhibited a high binding affinity for BPTF-665aa, characterized by minimal fluctuations at key interaction residues and robust hydrophobic and hydrogen bonding interactions, underscoring its potential as a selective inhibitor. The integration of virtual screening and MD simulations streamlined the drug discovery pipeline, reducing the time and resources needed for initial validation and optimizing compound selection. However, despite the promise demonstrated in computational modeling, experimental validation remains crucial. Future efforts will prioritize biochemical and cellular assays to assess HY-B0509’s impact on BPTF-665aa function, alongside in vivo investigations in T-LBL/ALL models [42]. Additionally, further structural optimization will be pursued to enhance its specificity, stability, and bioavailability, ultimately advancing HY-B0509 toward clinical translation [43].

In conclusion, this study provides the first demonstration that a circRNA-derived protein can regulate chromatin accessibility, broadening our understanding of circRNA functionality beyond miRNA sponging and transcriptional regulation. Our findings establish circBPTF and its encoded protein, BPTF-665aa, as central players in chemoresistance in T-LBL/ALL, unveiling a novel regulatory axis linking circRNA-derived proteins to chromatin remodeling. Furthermore, while our computational analyses identified HY-B0509 as a promising candidate, further experimental investigations are essential to determine its therapeutic applicability. Moving forward, this study provides a foundation for future research into circRNA-derived proteins as novel therapeutic targets, opening new avenues for addressing drug resistance in hematological malignancies.

## Supplementary Information

Supplementary Material 1.

Supplementary Material 2.

Supplementary Material 3.

Supplementary Material 4.

Supplementary Material 5.

Supplementary Material 6.

Supplementary Material 7.

Supplementary Material 8.

Supplementary Material 9.

Supplementary Material 10.

Supplementary Material 11.

Supplementary Material 12.

Supplementary Material 13.

Supplementary Material 14.

Supplementary Material 15.

Supplementary Material 16.

Supplementary Material 17.

Supplementary Material 18.

Supplementary Material 19.

Supplementary Material 20.

Supplementary Material 21.

Supplementary Material 22.

## Data Availability

ChIP-seq, ATAC-seq, and RNA-seq datasets for the K-562 cell line were retrieved from ChIP-Atla (https://chip-atlas.org), a comprehensive public repository for transcription factor binding profiles. Additionally, high-resolution Hi-C data (GSE237898; resolution) was downloaded from the NCBI GEO database (https://www.ncbi.nlm.nih.gov/geo/), with chromatin interaction maps visualized via the WashU Epigenome Browser (https://epigenomegateway.wustl.edu/browser/; hg38 assembly). The raw sequencing data underlying volcano plots in this study has been deposited in GEO under accession number GSE247590.
