# Supplementary material for: BPTF-665aa mediate chromatin remodeling drives chemoresistance in T-LBL/ALL
Source: J Exp Clin Cancer Res. 2025 Nov 7;44:302. doi: 10.1186/s13046-025-03556-8 (PMC12595860; doi:10.1186/s13046-025-03556-8)
Supplement: Supplementary file 1 — Supplementary Material 1. [file 13046_2025_3556_MOESM1_ESM.docx]

**Supplementary Figure 1. (A)** qRT-PCR quantitation of circBPTF expression in primary leukemic blasts from T-LBL/ALL patients. Significant upregulation was observed in the relapse group compared to the diagnostic group. Data are shown as mean ± SD (*n*=3). Student's *t*-test, **p* < 0.05. **(B)** Western blot analysis. BPTF-665aa intensity was elevated in the Relapse group. Full-length BPTF levels showed no significant difference between the relapse and diagnostic groups. **(C)** Expression levels of circBPTF across three T-cell malignancies as determined by qRT-PCR (*n*=3 per group). Cycle Threshold (Ct) values are shown on the Y-axis, with lower values indicating higher expression.

**Supplementary Figure 2.** Immunofluorescence analysis was performed to evaluate circBPTF expression and subcellular localization in primary patient samples. Nuclei were counterstained with DAPI (blue), while circBPTF was detected using specific antibodies conjugated with a red fluorescent dye. Scale bar: 10 μm.

**Supplementary Figure 3. (A)** Western blot confirms circBPTF knockdown by shRNA (shCirc-1/shCirc-2) specifically reduces BPTF-665aa protein. shNC (short hairpin negative control). **(B)** qPCR shows knockdown selectively suppresses circular but not linear expression. Data are shown as mean ± SD (*n*=3). Student's *t*-test, **p* < 0.05 shNC vs. shCirc-1; ^#^*p* < 0.05 shNC vs. shCirc-2. **(C)** Cell viability curves demonstrate enhanced sensitivity to Doxorubicin (Dox, 0.2 μM) in knockdown groups (shCirc-1/shCirc-2) compared to control (Ctrl). Data are shown as mean ± SD (*n*=3). Two-way ANOVA, **p* < 0.05 shNC vs. shCirc-1; ^#^*p* < 0.05 shNC vs. shCirc-2. **(D)** Flow cytometry verifies synergistic induction of apoptosis with Dox treatment. Data are shown as mean ± SD (*n*=3). Student's *t*-test, **p* < 0.05 shNC+Dox vs. shCirc-1+Dox; ^#^*p* < 0.05 shNC+Dox vs. shCirc-2+Dox.

**Supplementary Figure 4. (A)** Western blot confirms successful overexpression of WT and ORF BPTF-665aa protein. WT contains the full-length circBPTF sequence with an intact IRES and ORF; Mut includes a disrupted IRES region that prevents protein translation but permits circular RNA formation; ORF comprises the linear RNA sequence coding for the ORF, without generating circRNA. **(B)** qPCR indicates that BPTF-665aa overexpression upregulates circBPTF but not linear BPTF expression. Data are shown as mean ± SD (*n*=3). Student's *t*-test, **p* < 0.05 Ctrl vs. Mut; ^#^*p* < 0.05 Ctrl vs. WT. **(C)** Cell viability assays reveal that BPTF-665aa overexpression enhances sensitivity to Doxorubicin (Dox) treatment. Data are shown as mean ± SD (*n*=3). Two-way ANOVA, **p* < 0.05 Ctrl vs. WT; ^#^*p* < 0.05 Ctrl vs. ORF. **(D)** Flow cytometry demonstrates that BPTF-665aa overexpression synergizes with Dox to promote apoptosis. Data are shown as mean ± SD (*n*=3). Student's *t*-test, **p* < 0.05 Ctrl+Dox vs. WT+Dox; ^#^*p* < 0.05 Ctrl+Dox vs. ORF+Dox.

**Supplementary Figure 5.** BPTF-665aa-overexpressing cells (BPTF-665aa-OE) exhibit significantly reduced cell death compared to control cells (Ctrl) after treatment with **(A)** dexamethasone (DXM), **(B)** L-asparaginase (L-ASP), **(C)** vincristine (VCR), or **(D)** daunorubicin (DNR). For each drug, the left panel shows cell viability curves, and the right panel shows representative flow cytometry plots of apoptosis with corresponding quantitative analysis. Data are expressed as mean ± SD (*n* = 3). Statistical significance was determined using two-tailed Student's *t*-tests. Analyses of dose-response curves were conducted using two-way ANOVA. Statistical significance is presented as **p* < 0.05.

**Supplementary Figure 6. (A)** Western blot analysis of BPTF-665aa protein expression in high versus low expression groups of cells. **(B)** qRT-PCR analysis of target gene expression in high versus low expression groups of cells. Student's *t*-test, **p* < 0.05. **(C)** Analysis of cancer cell viability. Compared to the high expression group, low expression group significantly reduced cell survival after treatment. Before treatment, both groups showed high viability with a slight reduction in diagnostic group. Student's *t*-test, **p* < 0.05. **(D)** Transplantation survival analysis in a xenograft mouse model. Beginning on day 20 post-inoculation, tumor-bearing mice received intraperitoneal injections of Doxorubicin (Dox) every third day for two weeks.

**Supplementary Figure 7.** Cycloheximide (CHX) chase assay shows that circBPTF knockdown delays BPTF protein degradation. Data are shown as mean ± SD (*n*=3). Two-way ANOVA, **p* < 0.05.

**Supplementary Figure 8. (A)** BPTF enrichment was significantly higher in relapsed samples compared to diagnostic samples. Data are shown as mean ± SD (*n*=3). Student's *t*-test, **p* < 0.05. **(B)** Enhanced MYC-associated genomic signals at chromosome 8q24.21 in drug-resistant versus sensitive samples. Genomic profiling of the 8q24.21 region (chr8:127,720,646-127,760,512) reveals significantly elevated signal intensity in drug-resistant samples compared to drug-sensitive samples. The differential signals are localized within the MYC gene locus (RefSeq annotation shown below), suggesting potential MYC-related genomic alterations associated with drug resistance.

**Supplementary Figure 9. (A)** Western blot shows that BPTF-665aa overexpression upregulates c-Myc protein expression. **(B)** Bar graph showing that BPTF-665aa overexpression significantly increases *c-Myc* expression compared to the NC group. Data are shown as mean ± SD (*n*=3). Student's *t*-test, **p* < 0.05.

**Supplementary Figure 10. (A/B)** Changes in fold enrichment of H3K4me3 and H3K27ac in SUP-T1/K-562 cells under four genetic manipulations reveal mark-specific regulatory patterns. Data are shown as mean ± SD (*n*=3). Student's *t*-test, **p* < 0.05 Ctrl vs. WT; ^#^*p* < 0.05 Ctrl vs. ORF.

**Supplementary Figure 11. (A)** Temperature equilibration profile (290-310 K) with <1.5% fluctuation after 20 ns. **(B)** Pressure convergence (±400 bar dynamic range) achieving stability at 35 ns. **(C)** Density maintenance (1003±8 kg·m^-3^) matching aqueous system expectations. **(D)** Potential energy trajectory (-296,500±2,500 kJ·mol^-1^) confirming thermodynamic balance. **(E)** Total energy conservation (-239,000±3,000 kJ·mol^-1^) validating NVT ensemble controls. **(F)** Radius of gyration (1.45±0.03 nm) demonstrating structural consistency.

**Supplementary Figure 12. (A)** Cell viability curves of SUP-T1 and K-562 cells expressing ORF (constructs that produce only the protein product without forming circRNA) treated with increasing concentrations of Doxorubicin (Dox, 0.01-0.5 μM), with or without 5 μM inhibitor. Data are shown as mean ± SD (*n*=3). Two-way ANOVA, **p* < 0.05. **(B)** Flow cytometry analysis of apoptosis in SUP-T1 and K-562 cells by Annexin V-FITC/PI double staining. Bar graph shows quantitative analysis of total apoptotic cells. Data are shown as mean ± SD (*n*=3). Student's *t*-test, **p* < 0.05.

**Supplementary Figure 13.** Expression levels of circBPTF across different genetic backgrounds in the SYSUCC cohort (Total *n* = 140). The box plot shows the expression distribution in tissues without NOTCH1/FBXW7 mutations (white, *n* = 85), tissues with NOTCH1 mutations (red, *n* = 37), and tissues with FBXW7 mutations (blue, *n* = 18). The Y-axis represents Cycle Threshold (Ct) values. Lower Ct values indicate higher circBPTF expression.

**Supplementary Figure 14.** Representative full membrane corresponding to the protein bands shown in Figure 4A.

**Supplementary Figure 15.** Representative full membrane corresponding to the protein bands shown in Figure 4B.

**Supplementary Figure 16.** Representative full membrane corresponding to the protein bands shown in Supplementary Figure 1B.

**Supplementary Figure 17.** Representative full membrane corresponding to the protein bands shown in Supplementary Figure 6A.

**Supplementary Figure 18.** Representative full membrane corresponding to the protein bands shown in Supplementary Figure 7.
