# Supplementary figures and images for: BPTF-665aa mediate chromatin remodeling drives chemoresistance in T-LBL/ALL
Source: J Exp Clin Cancer Res. 2025 Nov 7;44:302. doi: 10.1186/s13046-025-03556-8 (PMC12595860; doi:10.1186/s13046-025-03556-8)

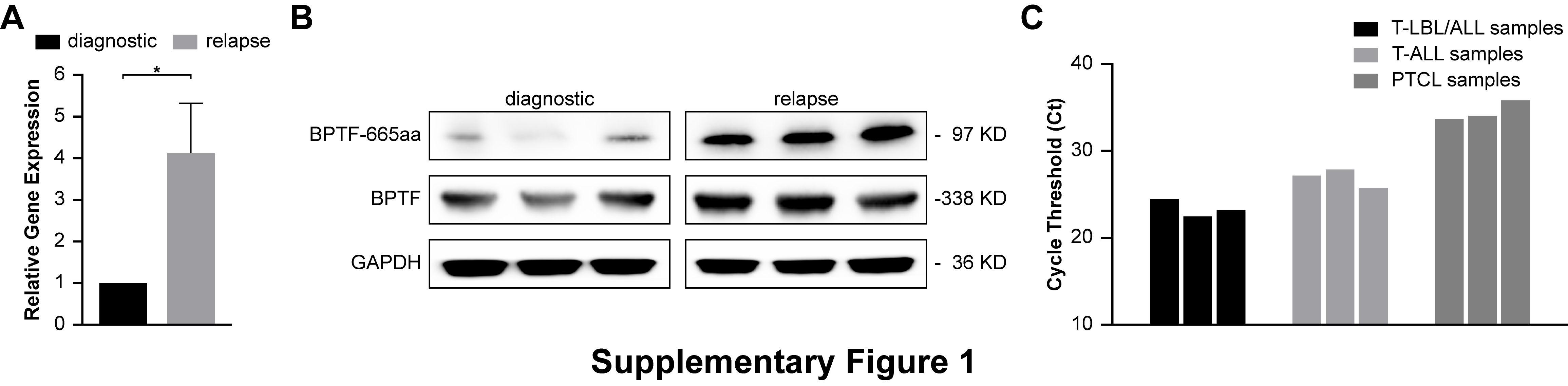

Supplement: Supplementary file 2 — Supplementary Material 2. [file 13046_2025_3556_MOESM2_ESM.tif]

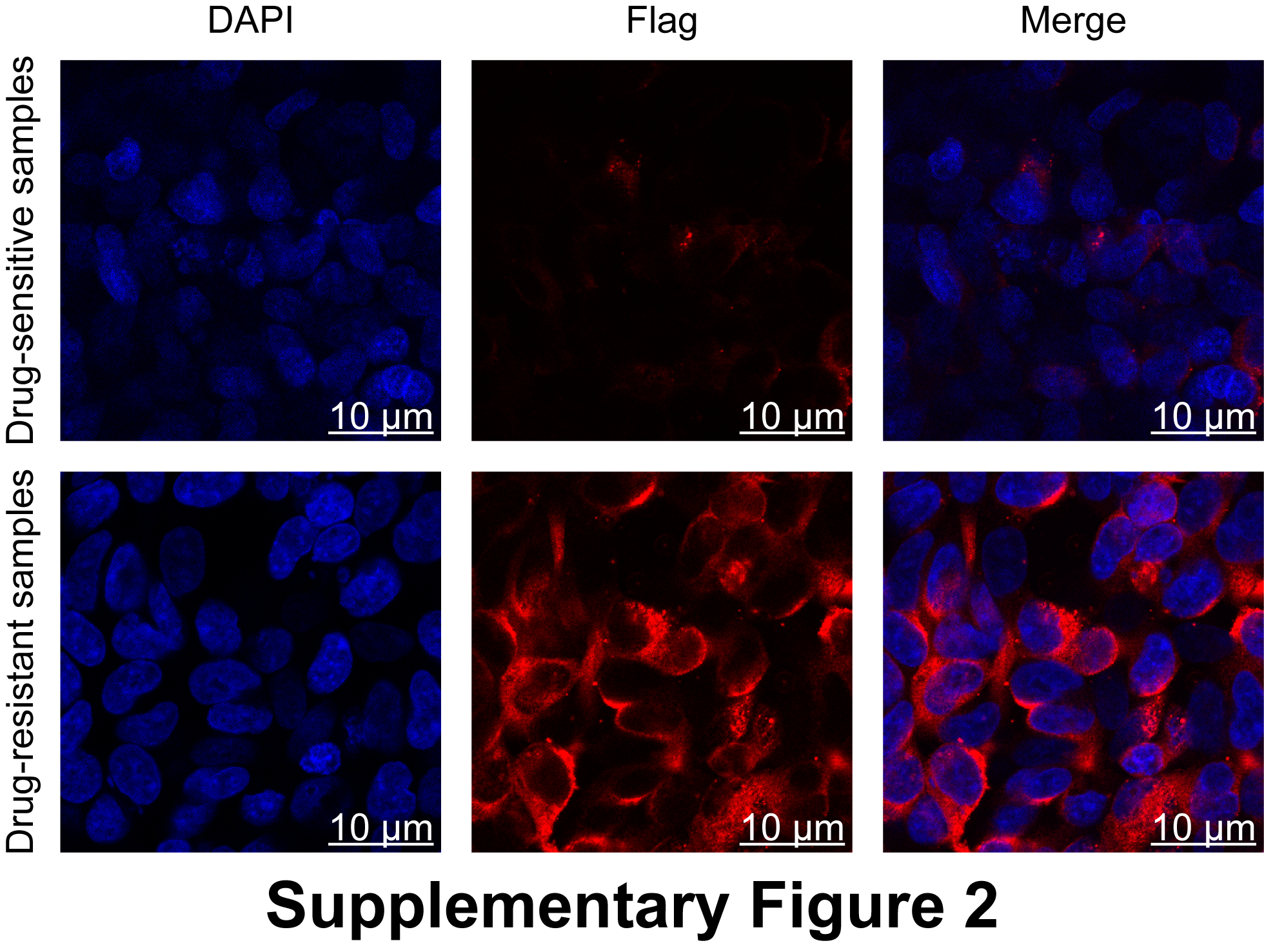

Supplement: Supplementary file 4 — Supplementary Material 4. [file 13046_2025_3556_MOESM4_ESM.tif]

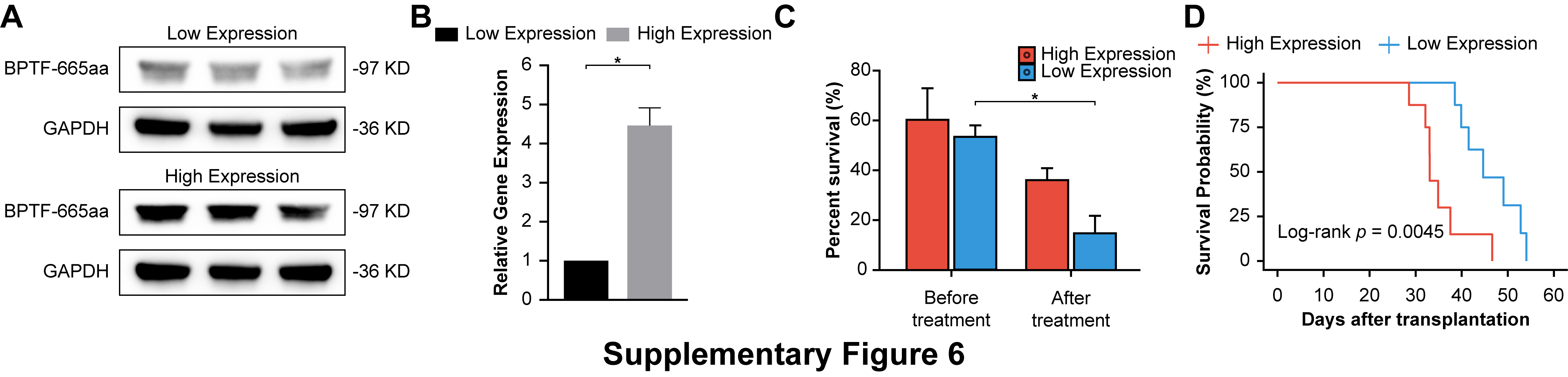

Supplement: Supplementary file 5 — Supplementary Material 5. [file 13046_2025_3556_MOESM5_ESM.tif]

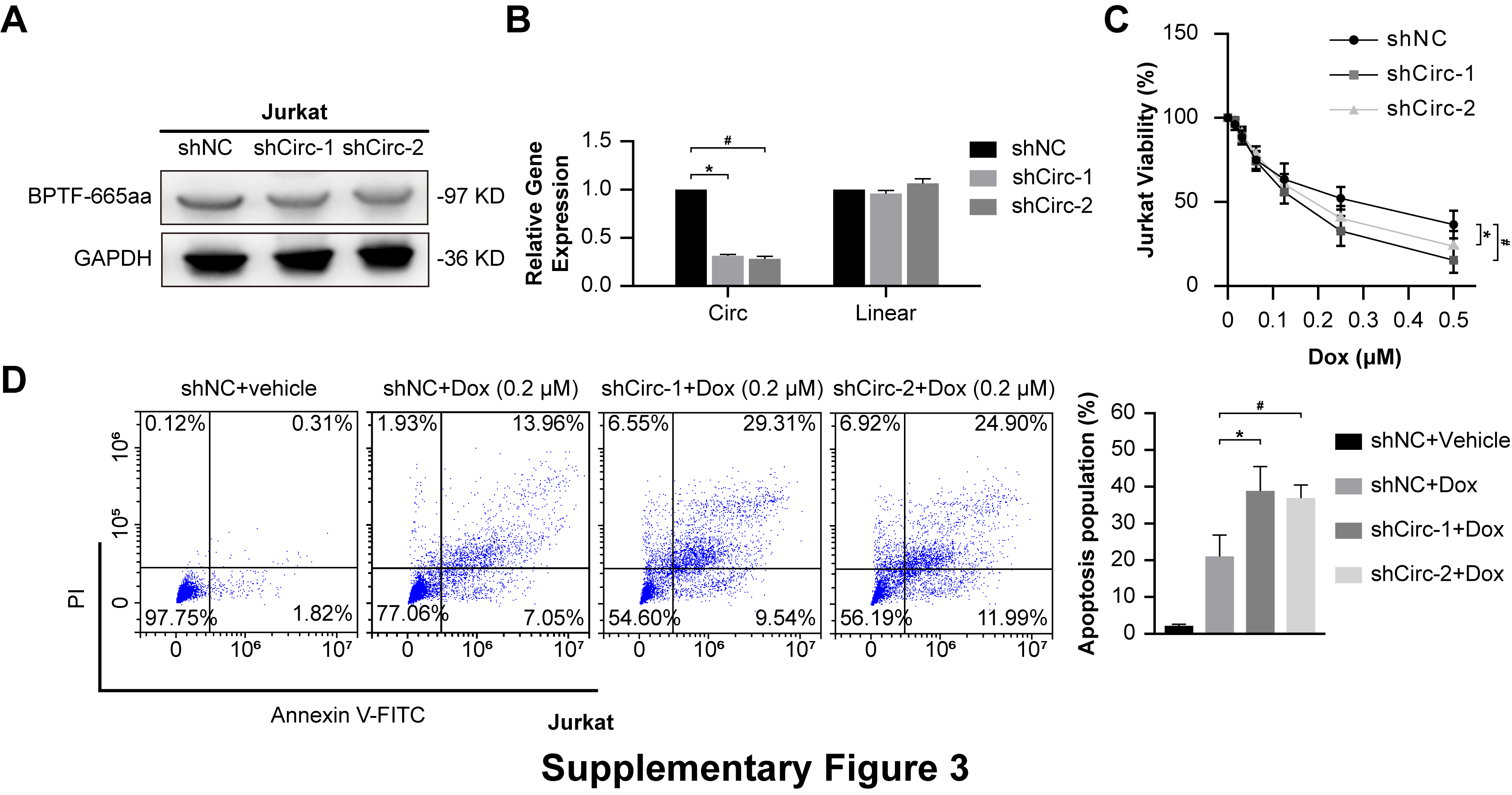

Supplement: Supplementary file 6 — Supplementary Material 6. [file 13046_2025_3556_MOESM6_ESM.tif]

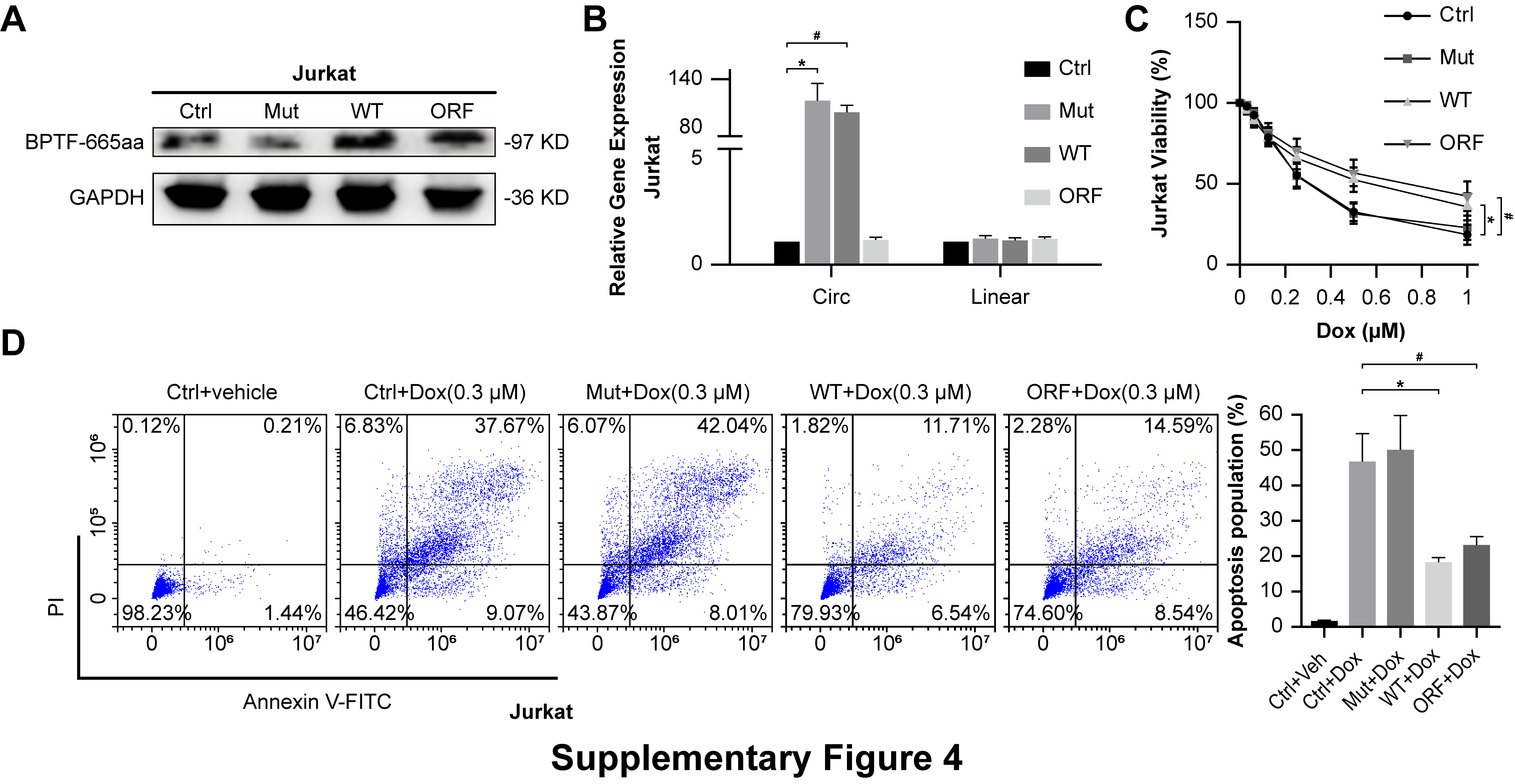

Supplement: Supplementary file 7 — Supplementary Material 7. [file 13046_2025_3556_MOESM7_ESM.tif]

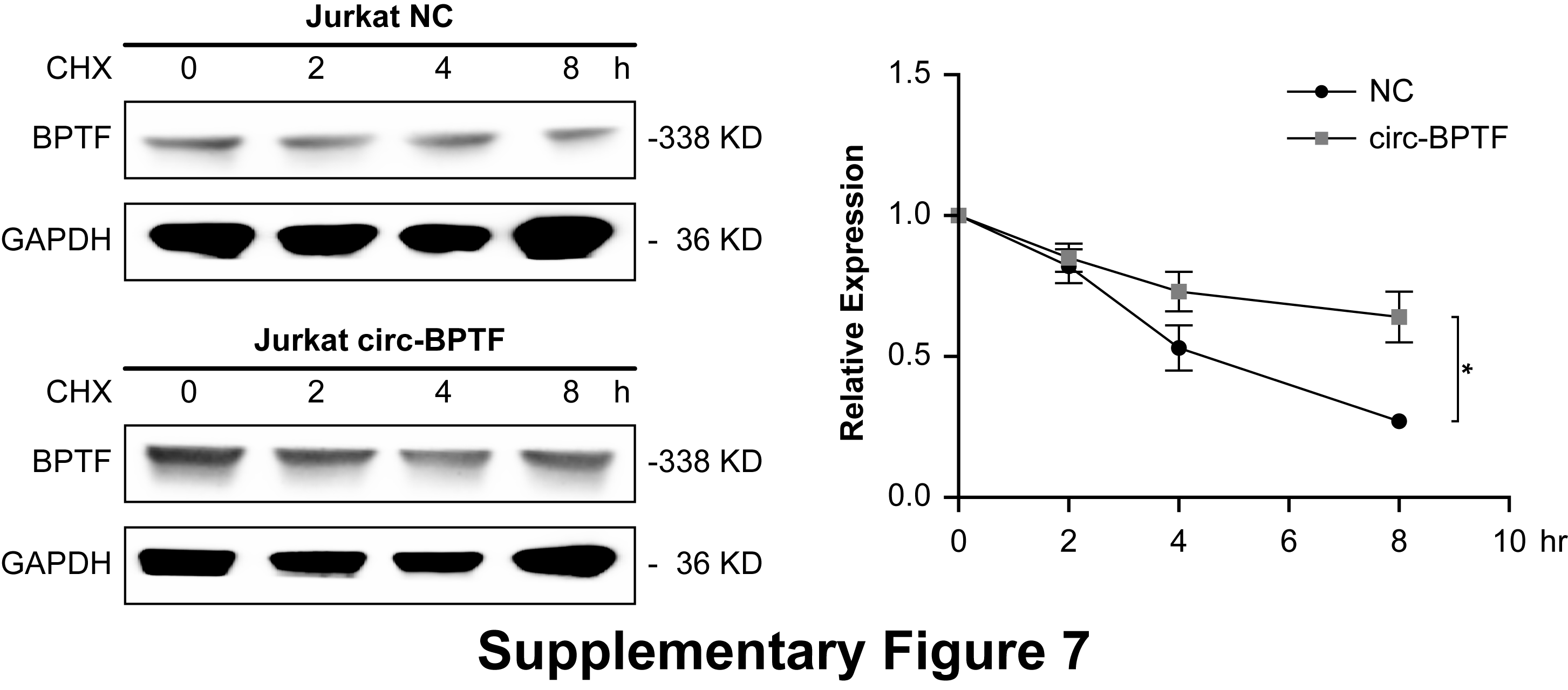

Supplement: Supplementary file 8 — Supplementary Material 8. [file 13046_2025_3556_MOESM8_ESM.tif]

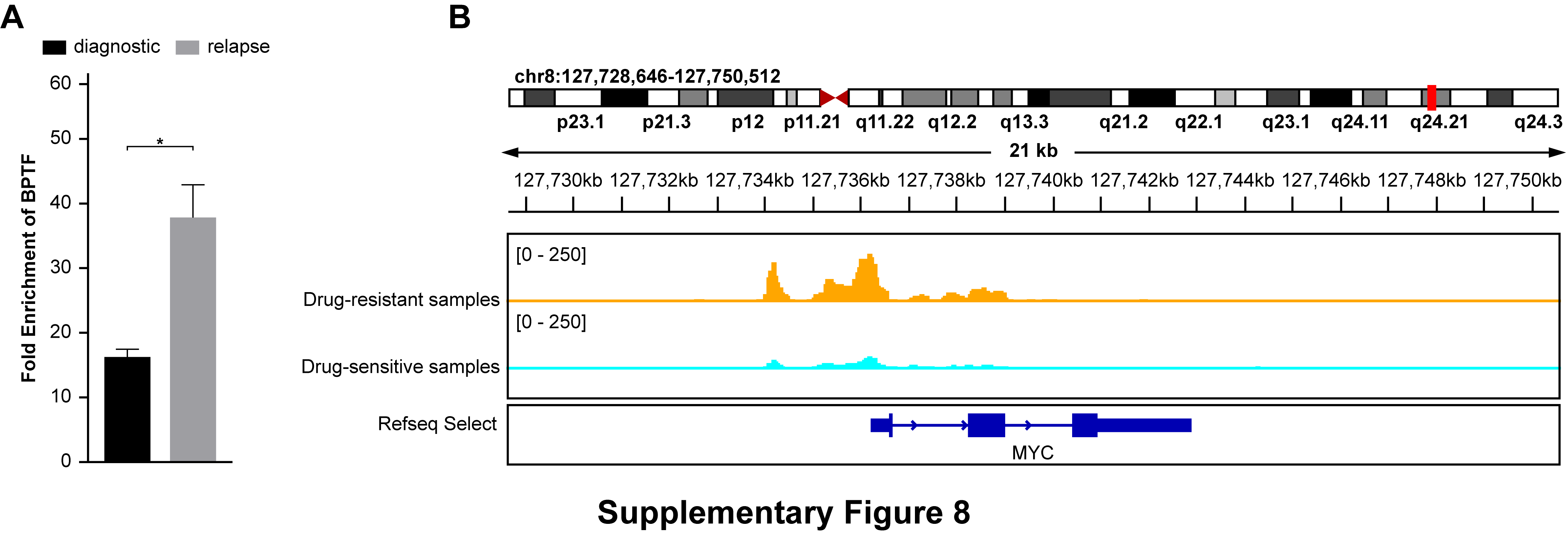

Supplement: Supplementary file 9 — Supplementary Material 9. [file 13046_2025_3556_MOESM9_ESM.tif]

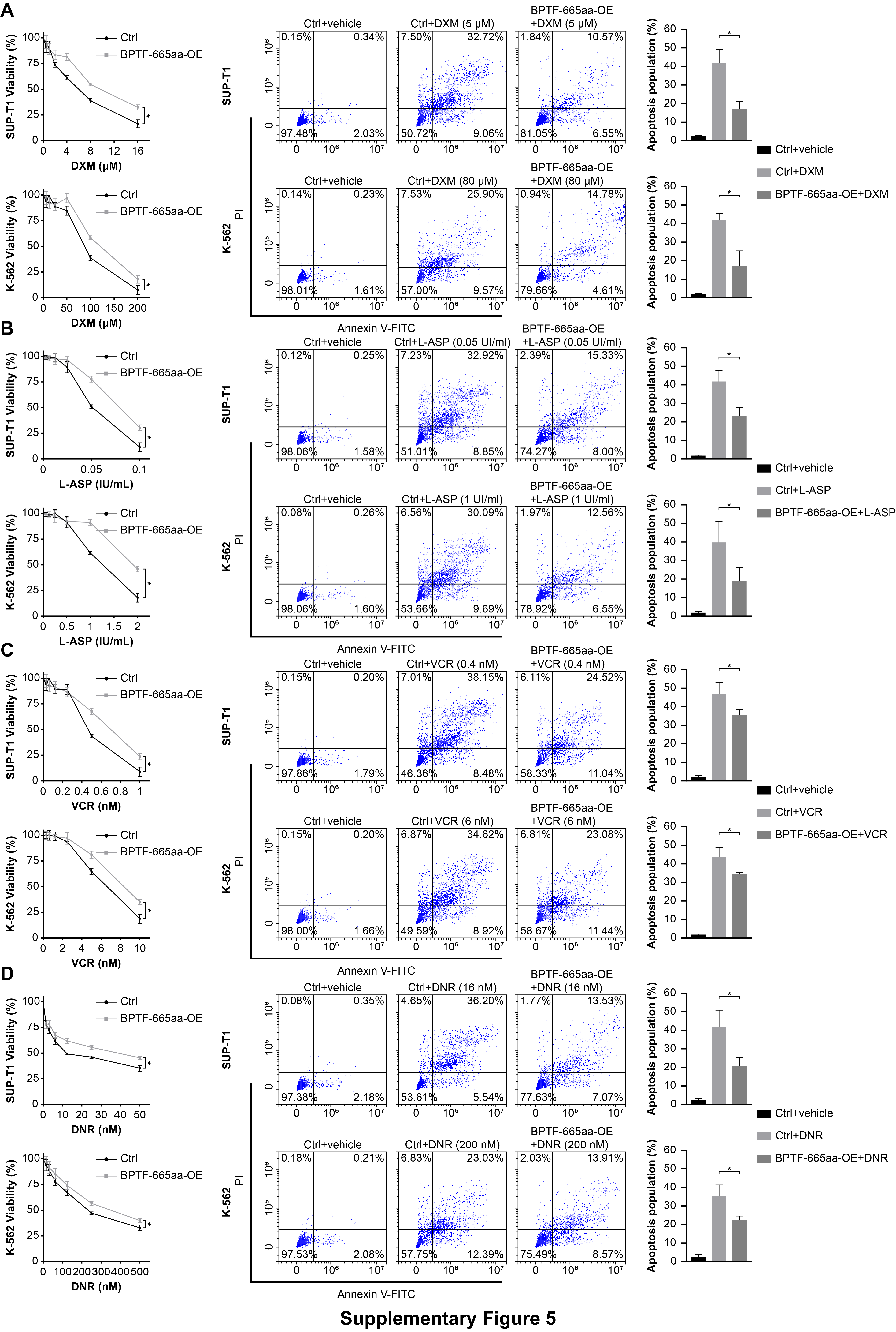

Supplement: Supplementary file 10 — Supplementary Material 10. [file 13046_2025_3556_MOESM10_ESM.tif]

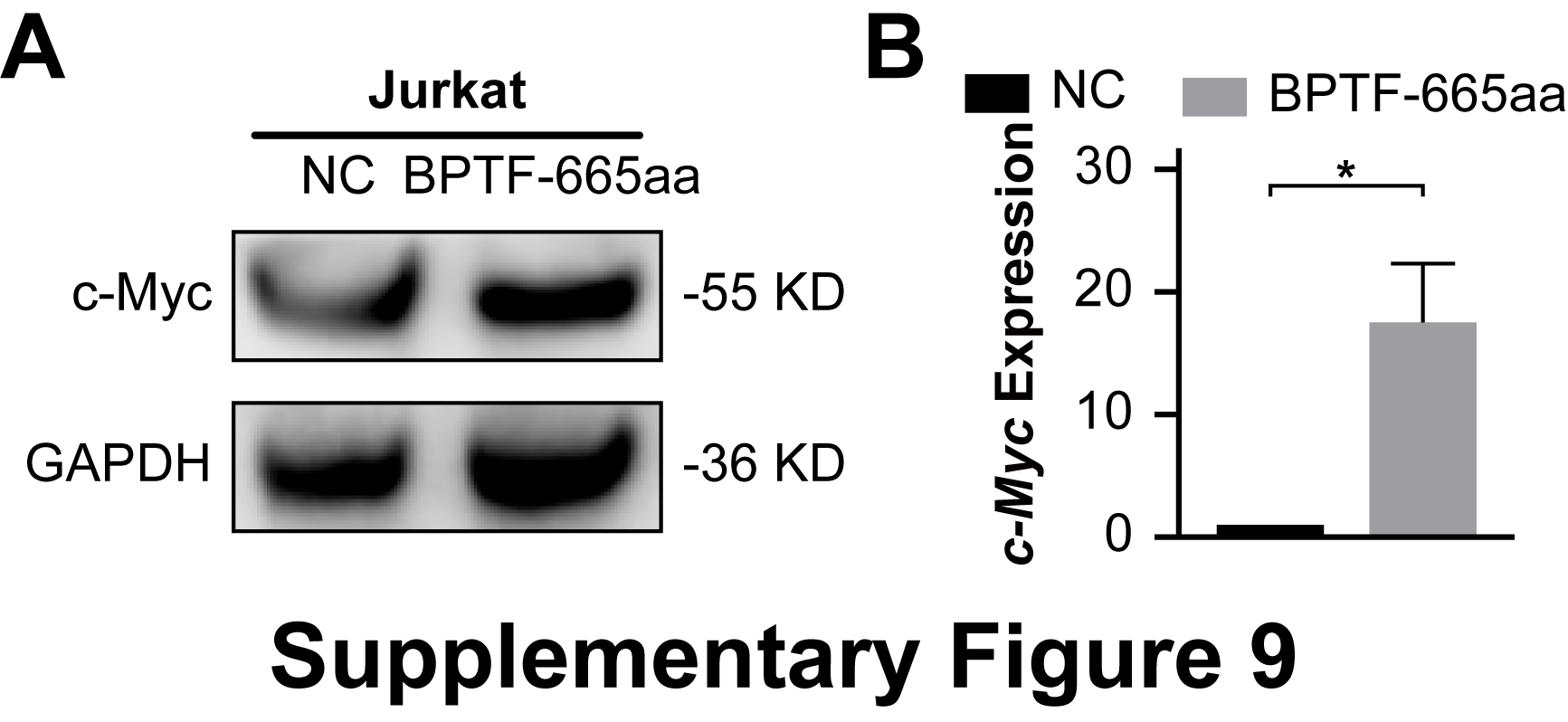

Supplement: Supplementary file 11 — Supplementary Material 11. [file 13046_2025_3556_MOESM11_ESM.tif]

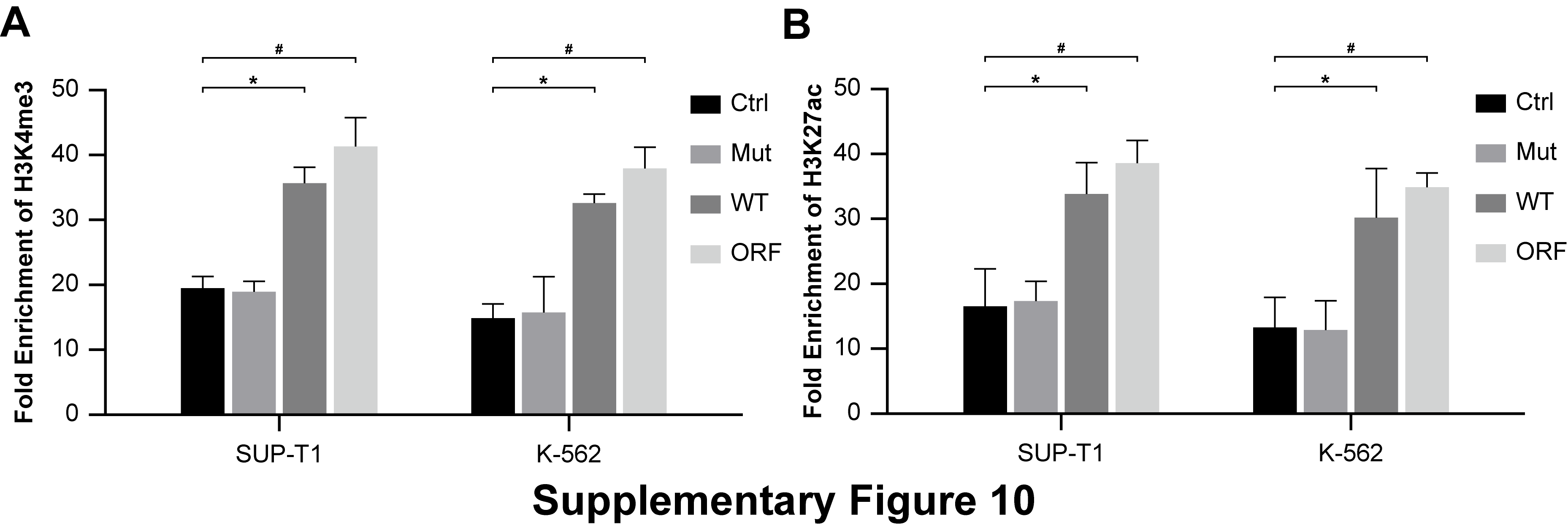

Supplement: Supplementary file 12 — Supplementary Material 12. [file 13046_2025_3556_MOESM12_ESM.tif]

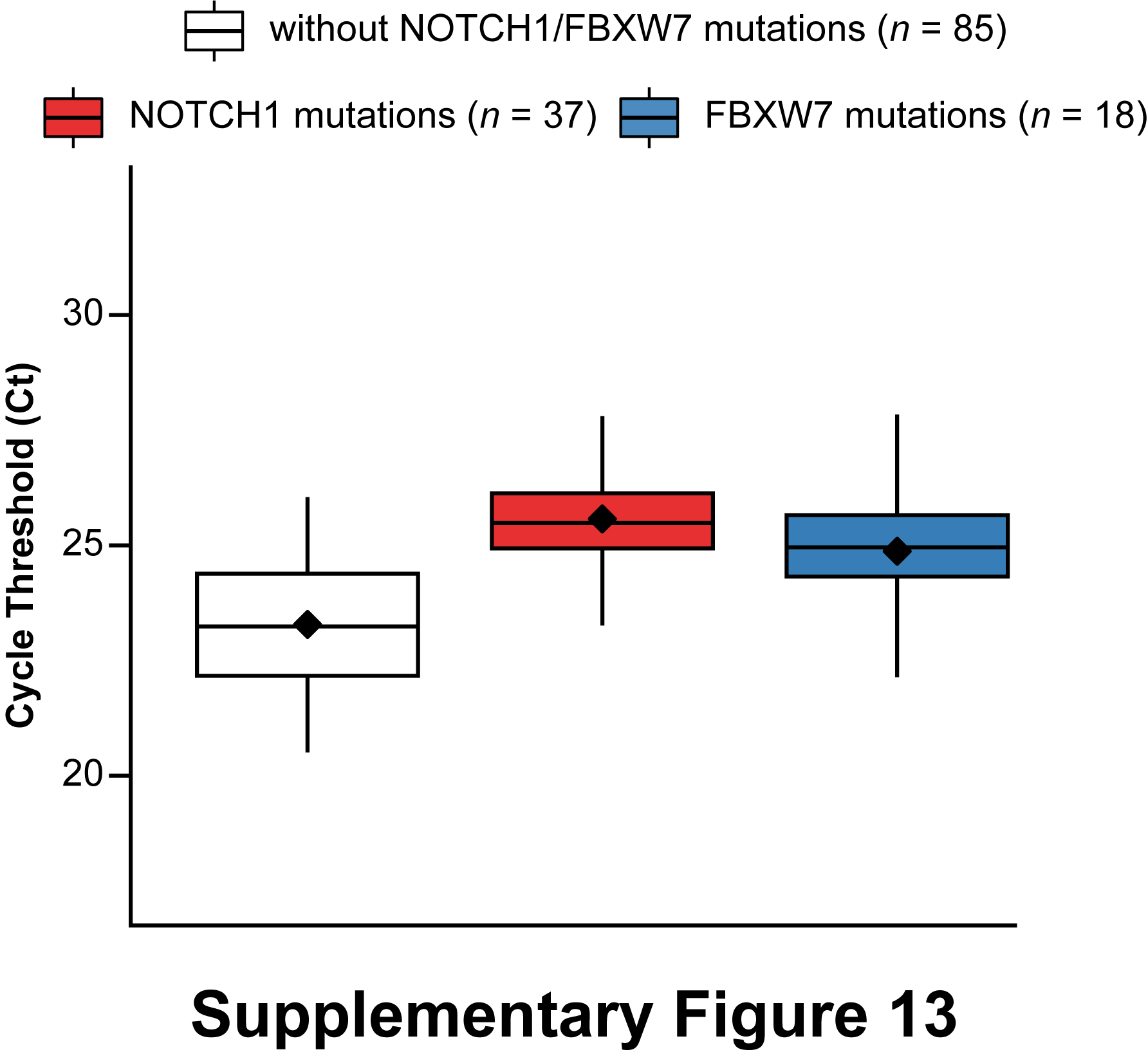

Supplement: Supplementary file 13 — Supplementary Material 13. [file 13046_2025_3556_MOESM13_ESM.tif]

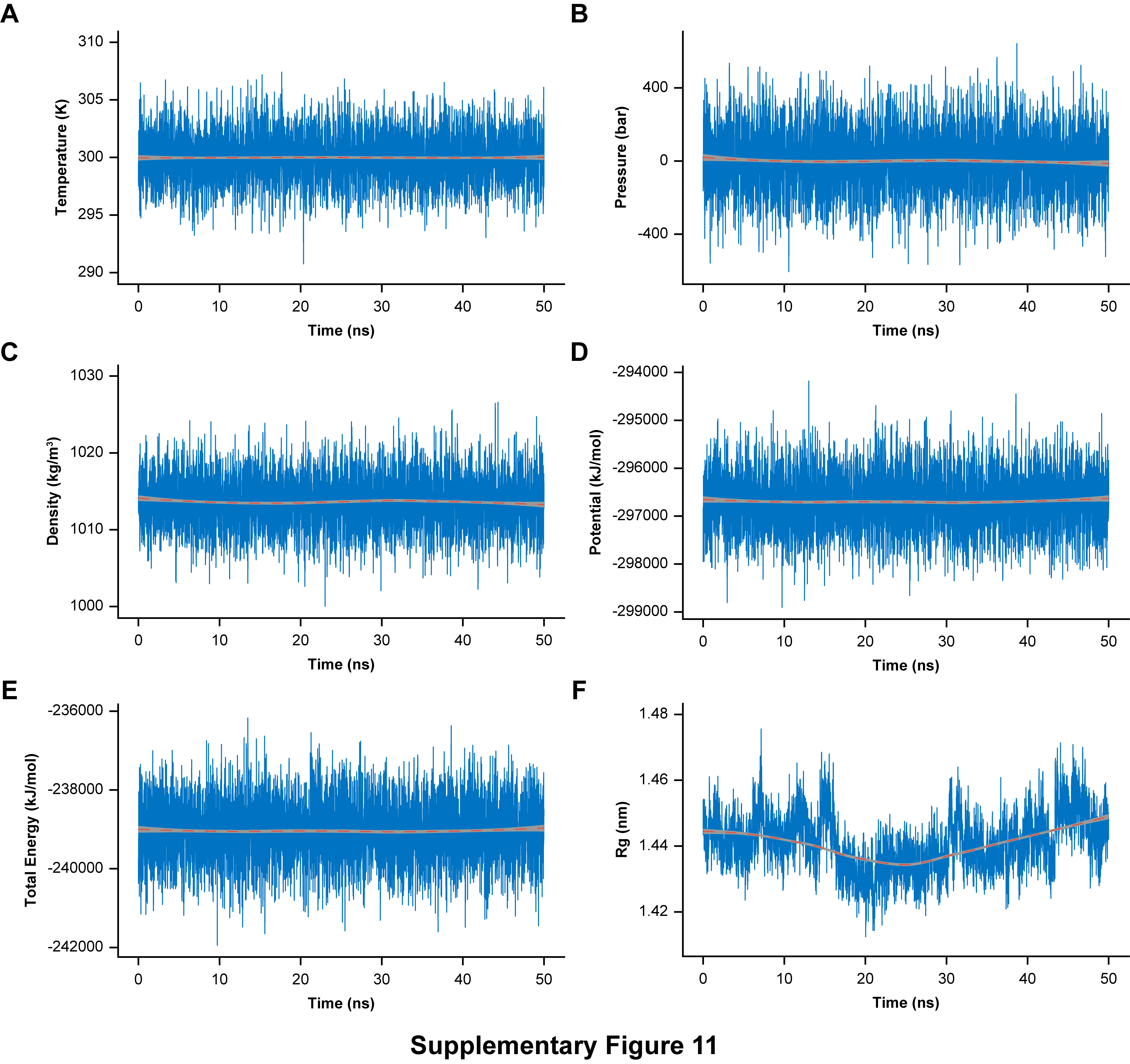

Supplement: Supplementary file 14 — Supplementary Material 14. [file 13046_2025_3556_MOESM14_ESM.tif]

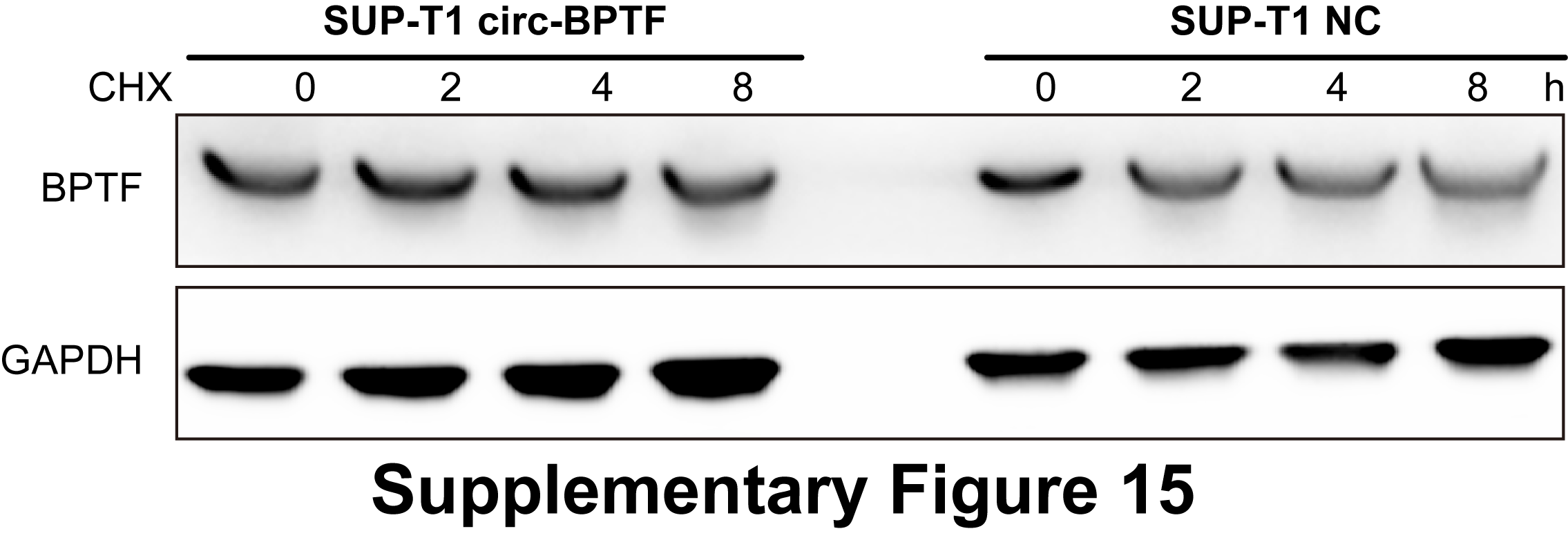

Supplement: Supplementary file 15 — Supplementary Material 15. [file 13046_2025_3556_MOESM15_ESM.tif]

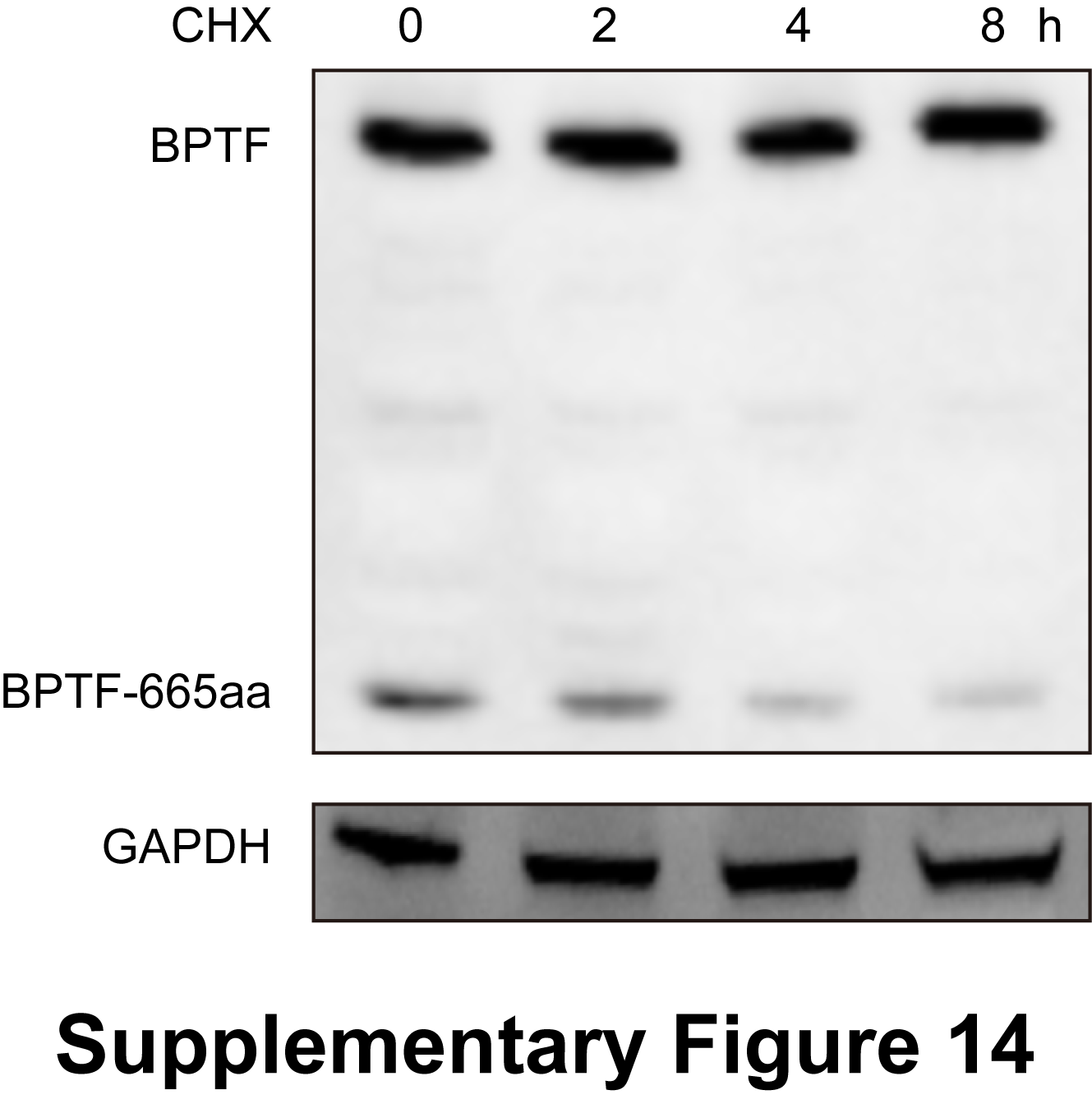

Supplement: Supplementary file 16 — Supplementary Material 16. [file 13046_2025_3556_MOESM16_ESM.tif]

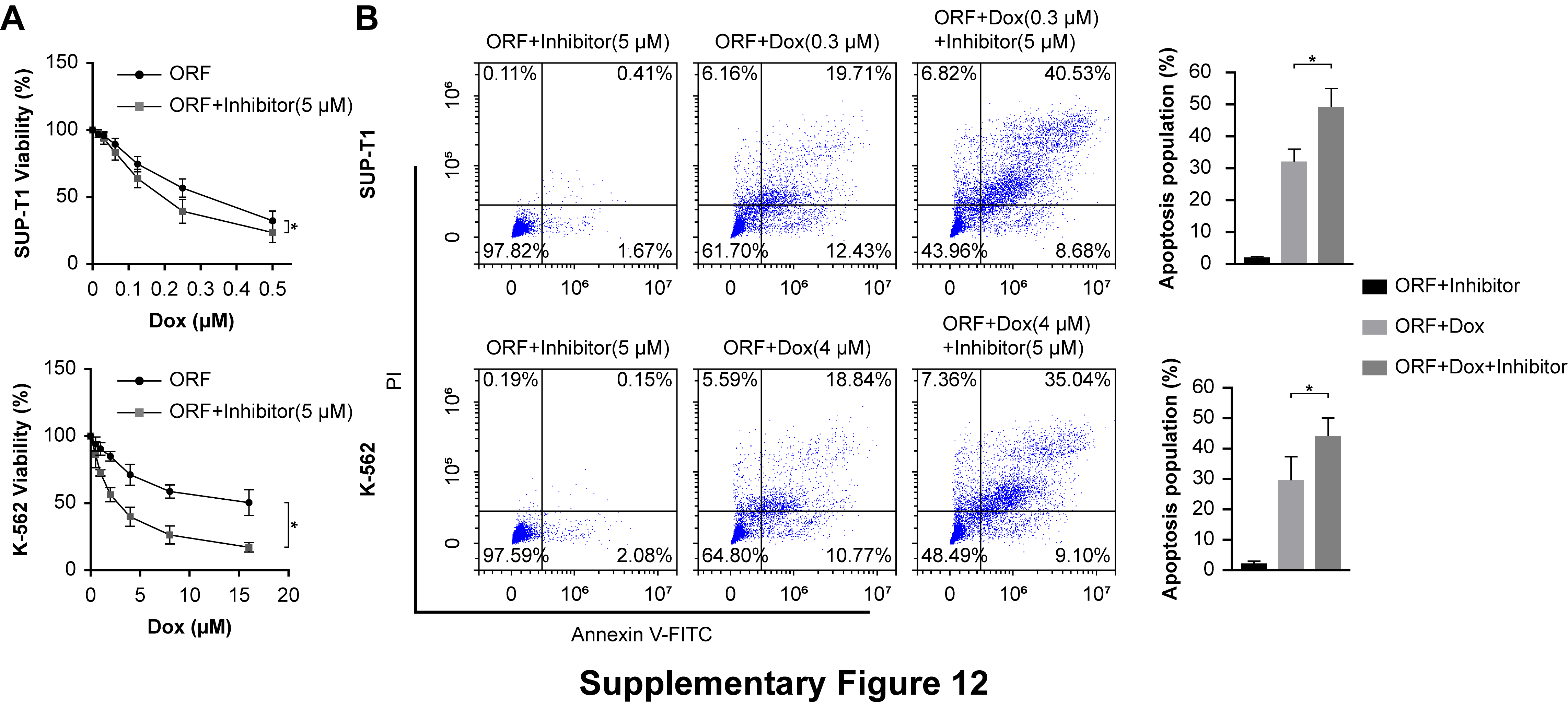

Supplement: Supplementary file 17 — Supplementary Material 17. [file 13046_2025_3556_MOESM17_ESM.tif]

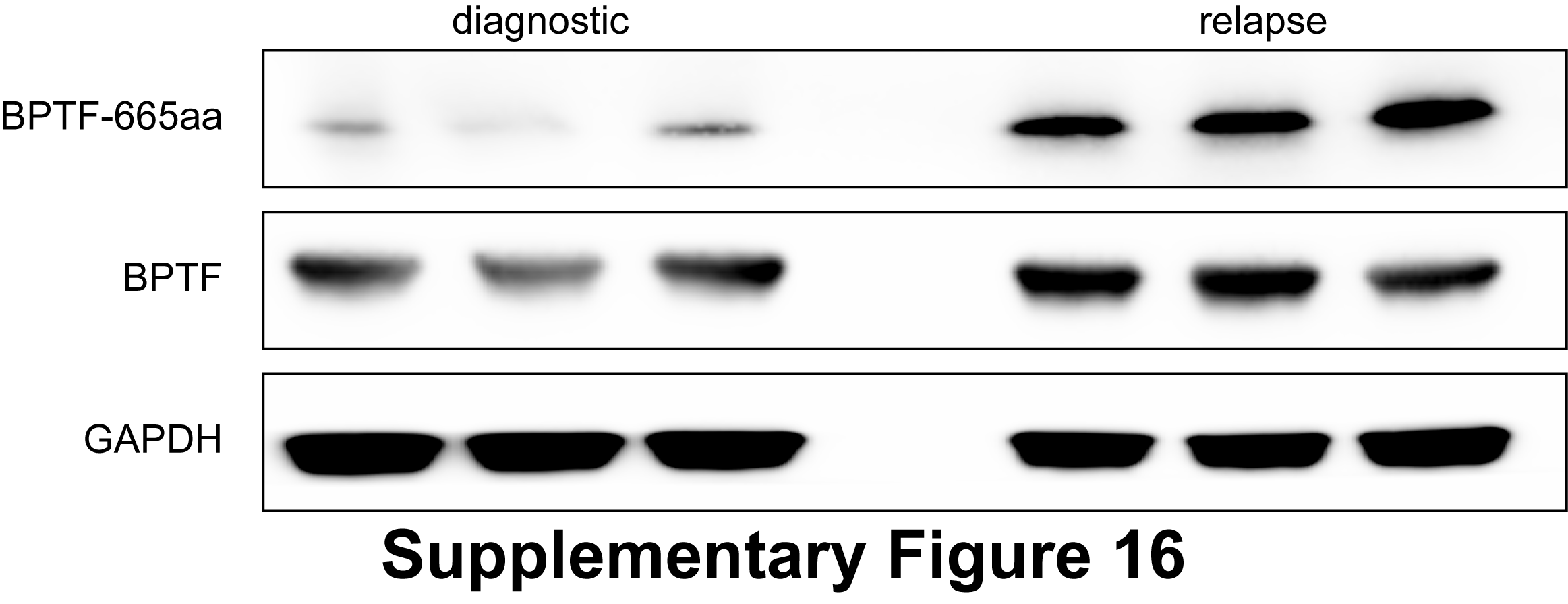

Supplement: Supplementary file 18 — Supplementary Material 18. [file 13046_2025_3556_MOESM18_ESM.tif]

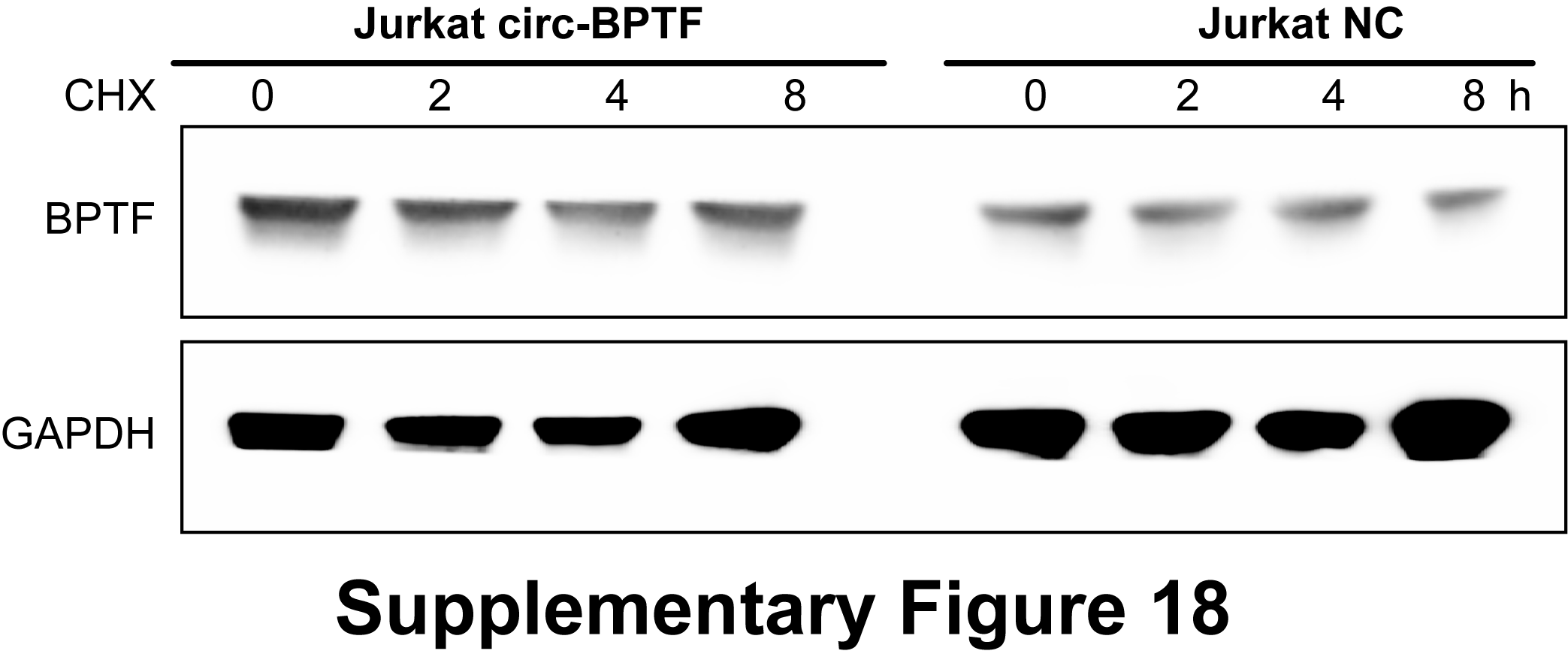

Supplement: Supplementary file 19 — Supplementary Material 19. [file 13046_2025_3556_MOESM19_ESM.tif]

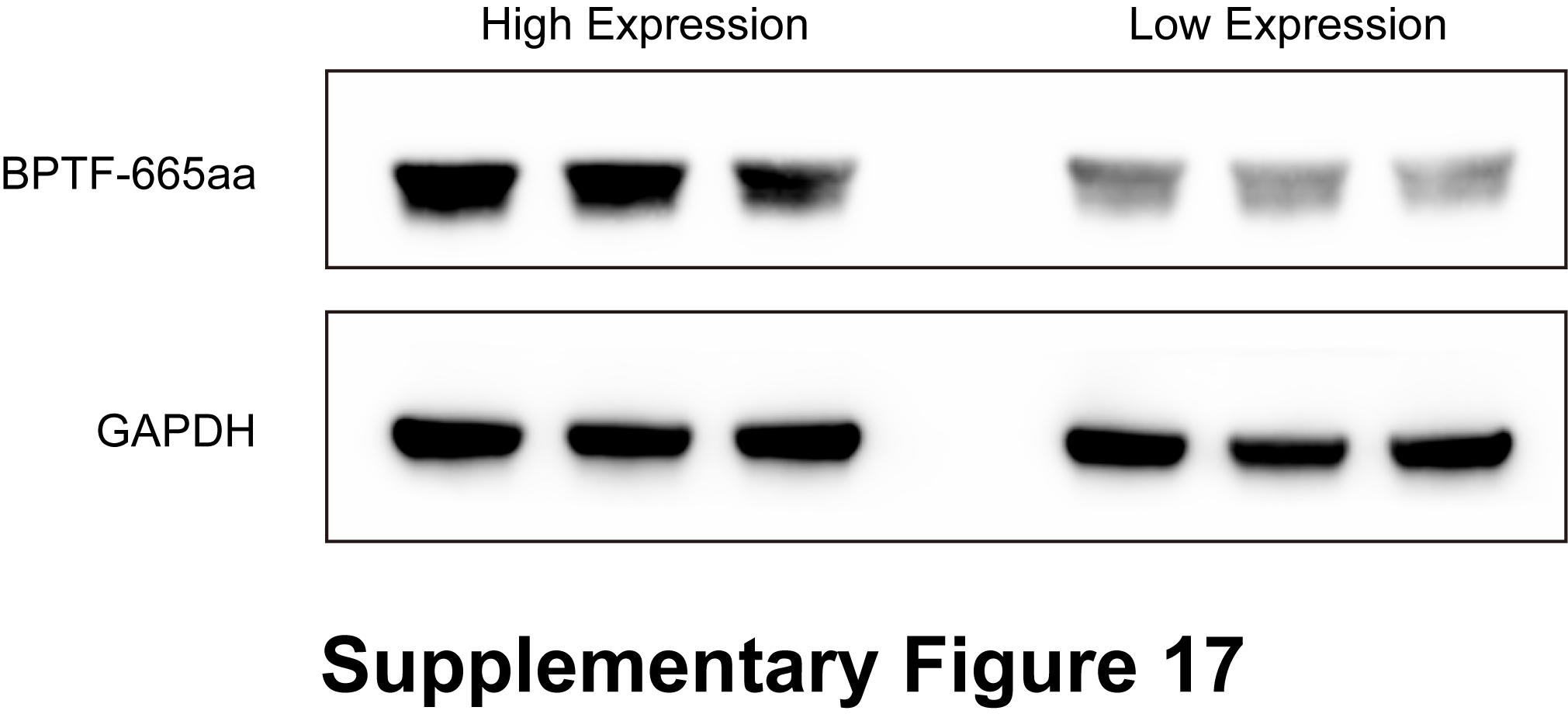

Supplement: Supplementary file 21 — Supplementary Material 21. [file 13046_2025_3556_MOESM21_ESM.tif]
