# Supplementary material for: BPTF-665aa mediate chromatin remodeling drives chemoresistance in T-LBL/ALL
Source: J Exp Clin Cancer Res. 2025 Nov 7;44:302. doi: 10.1186/s13046-025-03556-8 (PMC12595860; doi:10.1186/s13046-025-03556-8)
Supplement: Supplementary file 3 — Supplementary Material 3. [file 13046_2025_3556_MOESM3_ESM.docx]

Supplementary Table 1. Clinicopathologic Characteristics of T-cell lymphoblastic lymphoma (T-LBL) patients Stratified by circRNA.3579 Expression in the Training and Validation Set

| **Variable** | **Total cases** | **Training Set** | | **Total cases** | **Validation set** | |
| --- | --- | --- | --- | --- | --- | --- |
|  |  | High Expression of circRNA.3579 | Low Expression of circRNA.3579 |  | High Expression of circRNA.3579 | Low Expression of circRNA.3579 |
| **Age（years**） | | | | | | |
| ＞45 | 76 | 57 | 19 | 10 | 8 | 2 |
| ≤45 | 16 | 11 | 5 | 36 | 26 | 10 |
| **Gender** |  | | | | | |
| Male | 68 | 49 | 19 | 33 | 25 | 8 |
| Female | 24 | 19 | 5 | 13 | 9 | 4 |
| **ECOG PS** | | | | | | |
| <2 | 80 | 60 | 20 | 38 | 28 | 10 |
| ≥2 | 12 | 8 | 4 | 8 | 6 | 2 |
| **Effusion, pleural and/or pericardia** | | | | | | |
| Yes | 63 | 50 | 13 | 30 | 25 | 5 |
| No | 29 | 18 | 11 | 16 | 9 | 7 |
| **Mediastinal involvement** | | | | | | |
| Yes | 82 | 59 | 23 | 42 | 32 | 10 |
| No | 10 | 9 | 1 | 4 | 2 | 2 |
| **Bone marrow involvement** | | | | | | |
| Yes | 28 | 18 | 10 | 13 | 9 | 4 |
| No | 64 | 50 | 14 | 33 | 25 | 8 |
| **CNS involvement** | | | | | | |
| Yes | 3 | 2 | 1 | 4 | 3 | 1 |
| No | 89 | 66 | 23 | 42 | 31 | 11 |
| **LDH concertation** | | | | | | |
| Normal | 30 | 22 | 8 | 18 | 14 | 4 |
| Elevated | 62 | 46 | 16 | 28 | 20 | 8 |
| **Ann Arbor Stage** | | | | | | |
| ≤2 | 12 | 10 | 2 | 7 | 5 | 2 |
| ＞2 | 80 | 58 | 22 | 39 | 29 | 10 |
| **Relapse** |  | | | | | |
| Yes | 51 | 46 | 5 | 29 | 24 | 5 |
| No | 41 | 22 | 19 | 17 | 10 | 7 |

T-LBL: T-cell lymphoblastic lymphoma; ECOG-PS: Eastern Cooperative Oncology Group performance status; CNS: central nervous system; LDH: lactate dehydrogenase.
