# Supplementary material for: BPTF-665aa mediate chromatin remodeling drives chemoresistance in T-LBL/ALL
Source: J Exp Clin Cancer Res. 2025 Nov 7;44:302. doi: 10.1186/s13046-025-03556-8 (PMC12595860; doi:10.1186/s13046-025-03556-8)
Supplement: Supplementary file 20 — Supplementary Material 20. [file 13046_2025_3556_MOESM20_ESM.docx]

**Supplementary Table 2. Top 50 Compounds from MCE 50K Diversity and Bioactive Libraries Based on Docking Scores**

| **Structure** | **Catalog No** | **Name** | **Docking score** |
| --- | --- | --- | --- |
|  | HY-B0509 | Amikacin (hydrate) | -11.984 |
|  | HY-N0652 | 2,3,5,4'-Tetrahydroxystilbene 2-O-D-Glucoside | -11.728 |
|  | HY-125847 | Salvianolic acid F | -11.491 |
|  | HY-Q33269 | 4-((4-((furan-2-ylmethyl)amino)quinazolin-2-yl)amino)phenol | -11.159 |
|  | HY-13653 | (-)-Epigallocatechin Gallate | -11.146 |
|  | HY-A0006 | Pentostatin | -11.123 |
|  | HY-13605 | Cytarabine | -11.069 |
|  | HY-N0319 | Salvianolic acid C | -10.712 |
|  | HY-111558A | Bobcat339 (hydrochloride) | -10.653 |
|  | HY-Q42121 | (E)-2-((2-hydroxy-3-methoxybenzylidene)amino)-N-phenylbenzamide | -10.627 |
|  | HY-N0898A | (-)-Catechin | -10.609 |
|  | HY-Q30088 | (S)-(4-(3-(4-chloro-3,5-dimethyl-1H-pyrazol-1-yl)-2-hydroxypropoxy)phenyl)(phenyl)methanone | -10.597 |
|  | HY-Q09974 | 5-(((2,4-dimethylphenyl)amino)methyl)-2-(4-fluorophenyl)-[1,2,4]triazolo[1,5-a]pyrimidin-7(4H)-one | -10.536 |
|  | HY-150508 | MK-0159 | -10.535 |
|  | HY-14858 | Derenofylline | -10.507 |
|  | HY-Q09217 | 2-((2-oxo-2-((4-phenylthiazol-2-yl)amino)ethyl)thio)nicotinic acid | -10.480 |
|  | HY-11009 | CGP60474 | -10.405 |
|  | HY-10915 | N-(2-((3,4-dichlorophenyl)amino)quinolin-4-yl)cyclohexanecarboxamide | -10.400 |
|  | HY-10240 | Mericitabine | -10.396 |
|  | HY-100673 | LM22A-4 | -10.393 |
|  | HY-W282615 | Antibacterial agent 117 | -10.392 |
|  | HY-16382 | PCI-27483 | -10.392 |
|  | HY-Q09978 | 5-((4-(2-hydroxyethyl)piperazin-1-yl)methyl)-2-phenyl-[1,2,4]triazolo[1,5-a]pyrimidin-7(4H)-one | -10.381 |
|  | HY-N0578 | Apigenin 7-glucoside | -10.367 |
|  | HY-W012293 | 3'-Deoxycytidine | -10.353 |
|  | HY-N2008 | Luteolin 5-O-glucoside | -10.338 |
|  | HY-Q42603 | N-((4-phenylthiazol-2-yl)carbamothioyl)cyclobutanecarboxamide | -10.169 |
|  | HY-Q04026 | (S)-4-phenyl-N-(1-(3-phenyl-1H-pyrazol-5-yl)pyrrolidin-3-yl)butanamide | -10.150 |
|  | HY-125035 | (R)-N2-(3-methoxyphenyl)-N4-((tetrahydrofuran-2-yl)methyl)quinazoline-2,4-diamine | -10.102 |
|  | HY-N7653 | Azaleatin | -10.044 |
|  | HY-122611A | CSRM617 (hydrochloride) | -10.030 |
|  | HY-105268 | AzddMeC | -10.029 |
|  | HY-B2137 | S-(+)-Ketoprofen | -10.024 |
|  | HY-Q37075 | (E)-3-(benzylideneamino)-8-methoxy-2-methyl-3H-pyrimido[5,4-b]indol-4(5H)-one | -9.964 |
|  | HY-Q38244 | 3-(2-((4-fluorophenyl)thio)ethyl)quinazoline-2,4(1H,3H)-dione | -9.900 |
|  | HY-N3516 | Oxyresveratrol 2-O-D-Glucopyranoside | -9.872 |
|  | HY-141885 | APOL1-IN-1 | -9.869 |
|  | HY-103490 | Takinib | -9.837 |
|  | HY-Q04299 | 2-(4-chlorophenyl)-5-((4-ethylpiperazin-1-yl)methyl)-[1,2,4]triazolo[1,5-a]pyrimidin-7(4H)-one | -9.831 |
|  | HY-Q04909 | (R)-4-methyl-N-(4-(pyridin-3-yl)thiazol-2-yl)-2-(1H-pyrrol-1-yl)pentanamide | -9.807 |
|  | HY-Q30919 | N-(4-(4-fluorophenyl)thiazol-2-yl)-2-((4-fluorophenyl)thio)acetamide | -9.774 |
|  | HY-13518 | Piceatannol | -9.749 |
|  | HY-102023 | GNF351 | -9.734 |
|  | HY-W012009 | 2'-Deoxy-2'-fluorocytidine | -9.664 |
|  | HY-112113 | SLV-2436 | -9.651 |
|  | HY-19931 | COH29 | -9.636 |
|  | HY-Q43636 | 2-(4-(((4-chloro-3-(oxazolo[4,5-b]pyridin-2-yl)phenyl)amino)methyl)-2-methoxyphenoxy)acetamide | -9.630 |
|  | HY-Q26171 | tert-butyl 4-(3-phenyl-1H-pyrazol-5-yl)piperazine-1-carboxylate | -9.616 |
|  | HY-N6020A | (+)-Butin | -9.615 |
|  | HY-135981 | CMS-121 | -9.597 |
